# **Two-polarized roles of transcription factor FOSB in lung cancer progression and prognosis: dependent on p53 status**

**DOI:** 10.1186/s13046-024-03161-1

**Published:** 2024-08-21

**Authors:** Hongchao Zhang, Guopei Zhang, Mingyang Xiao, Su Cui, Cuihong Jin, Jinghua Yang, Shengwen Wu, Xiaobo Lu

**Affiliations:** 1 Key Laboratory of Environmental Stress and Chronic Disease Control & Prevention, Ministry of Education (China Medical University), Shenyang, 110122 People’s Republic of China; 2 Department of Toxicology, School of Public Health, China Medical University, No.77 Puhe Road, Shenyang North New District, Shenyang, 110122 People’s Republic of China; 3 Center of Gallstone Disease, Shanghai East Hospital & Institute of Gallstone Disease, School of Medicine, Tongji University, Shanghai, 200120 People’s Republic of China; 4 Department of Thoracic Surgery, Ward 2, The First Hospital of China Medical University, No.155 North Nanjing Street, Heping District, Shenyang, 110001 People’s Republic of China

**Keywords:** Lung cancer, Non-small cell lung cancer (NSCLC), Cisplatin sensitivity, Activator protein-1 (AP-1), FOSB, TP53, PREX1, IGFBP5, AKR1C3, ALDH3A1, MAPK/ERK, PI3K/AKT

## Abstract

**Background:**

Activator protein-1 (AP-1) represents a transcription factor family that has garnered growing attention for its extensive involvement in tumor biology. However, the roles of the AP-1 family in the evolution of lung cancer remain poorly characterized. FBJ Murine Osteosarcoma Viral Oncogene Homolog B (FOSB), a classic AP-1 family member, was previously reported to play bewilderingly two-polarized roles in non-small cell lung cancer (NSCLC) as an enigmatic double-edged sword, for which the reasons and significance warrant further elucidation.

**Methods and Results:**

Based on the bioinformatics analysis of a large NSCLC cohort from the TCGA database, our current work found the well-known tumor suppressor gene *TP53* served as a key code to decipher the two sides of FOSB – its expression indicated a positive prognosis in NSCLC patients harboring wild-type *TP53* while a negative one in those harboring mutant *TP53*. By constructing a panel of syngeneically derived NSCLC cells expressing p53 in different statuses, the radically opposite prognostic effects of FOSB expression in NSCLC population were validated, with the *TP53-R248Q* mutation site emerging as particularly meaningful. Transcriptome sequencing showed that FOSB overexpression elicited diversifying transcriptomic landscapes across NSCLC cells with varying genetic backgrounds of *TP53* and, combined with the validation by RT-qPCR, *PREX1* (*TP53-Null*), *IGFBP5* (*TP53-WT*), *AKR1C3*, and *ALDH3A1* (*TP53-R248Q*) were respectively identified as p53-dependent transcriptional targets of FOSB. Subsequently, the heterogenous impacts of FOSB on the tumor biology in NSCLC cells via the above selective transcriptional targets were confirmed in vitro and in vivo. Mechanistic investigations revealed that wild-type or mutant p53 might guide FOSB to recognize and bind to distinct promoter sequences via protein-protein interactions to transcriptionally activate specific target genes, thereby creating disparate influences on the progression and prognosis in NSCLC.

**Conclusions:**

FOSB expression holds promise as a novel prognostic biomarker for NSCLC in combination with a given genetic background of *TP53*, and the unique interactions between FOSB and p53 may serve as underlying intervention targets for NSCLC.

**Supplementary Information:**

The online version contains supplementary material available at 10.1186/s13046-024-03161-1.

## Background

Lung cancer continues to be the predominant burden on the occurrence and death of malignant tumors worldwide [1]. In clinical practice, lung cancer is more commonly (> 85%) diagnosed as non-small cell lung cancer (NSCLC), which consists primarily of lung adenocarcinoma (LUAD) and lung squamous cell carcinoma (LUSC) on the basis of histological classification [2]. Despite the inspiring progress in the knowledge of the disease biology of lung cancer and its management over the past few decades, the overall prognosis for NSCLC remains hardly sanguine, with a 5-year survival of 26% [3]. The high mortality of NSCLC is associated with its distinctly aggressive properties, with most cases diagnosed as advanced diseases [2]. For those patients (Stages III & IV) who are deprived of the opportunity for surgical ablation, chemotherapy is the preferred treatment strategy [4]. Cisplatin is widely used in post-operative adjuvant chemotherapy or systemic treatment for NSCLC due to its high responsiveness and economy. However, intrinsic or acquired platinum resistance in tumor cells has become a major cause of progression, recurrence, and death for NSCLC [5]. On this account, identifying key molecular events that drive the malignant progression and platinum resistance in lung cancer to screen for promising prognostic biomarkers or potential therapeutic targets, is of vital significance towards improving the prognosis of lung cancer.

Growing epidemiological evidence has revealed strong associations of the band 3, region 1, long arm of chromosome 19 (19q13) with the risk and platinum-based chemotherapy prognosis in NSCLC [6, 7]. FBJ Murine Osteosarcoma Viral Oncogene Homolog B (*FOSB*), a gene located at 19q13, encodes a protein that serves as a member of the Activator protein-1 (AP-1) transcription factor family. The AP-1 family has long been well known for its extensive involvement in multiple aspects of tumor biology, including oncogenic transformation, progression, and drug resistance [8]. Depending on specific histological type, differentiation status, or genetic background, AP-1 shows pro- or anti-tumor effects in flexibly variable forms of dimeric complexes as a double-edged sword [9]. The role of FOSB in the progression and prognosis of lung cancer seems extremely challenging to follow. A molecular epidemiological investigation showed that FOSB expression was significantly decreased in NSCLC tissues and correlated with an unfavorable prognosis in patients [10]. Tumor biology studies have indicated that FOSB-mediated transcriptional activation of the matrix metalloproteinase MMP-2 is involved in the invasion and metastasis in NSCLC driven by the pro-survival protein Bcl-2 [11]. Moreover, chemotherapeutic agent-induced FOSB expression might contribute to a counterproductively poor prognosis in NSCLC by endowing tumor cells with increased proliferation and invasiveness [12, 13]. Interestingly, however, several lines of other studies have yielded overwhelmingly opposite observations. It was reported that FOSB deficiency promoted the transformation and migration activity in NSCLC cells via the remodeling of intercellular junctions [14]. Furthermore, as a potential endogenous chemo-sensitizing factor, FOSB-mediated cell apoptosis and microtubule dynamics defects were shown to be involved in cisplatin- and doxorubicin-induced cytotoxicity in NSCLC, respectively [15–17]. Whether FOSB plays the role of a friend or foe in the evolution and prognosis of lung cancer? And how can we appropriately utilize the “two sides” of FOSB to optimize the management of lung cancer? For answers to the above questions, it is imperative to elucidate the critical molecular switch that controls the role reversal of FOSB, and the exact molecular mechanisms by which FOSB has radically opposite impacts on the progression and prognosis of lung cancer within defined contexts.

Tumor Protein P53 (*TP53*), a well-known tumor suppressor gene, is hailed as “Guardian of the genome” and has been widely used as a reliable biomarker for the susceptibility, diagnosis, and prognosis as well as a promising therapeutic target in most human tumors for its far-reaching influences on tumor biology. The p53 protein, encoded by the *TP53* gene, is activated via multiple pathways in response to a variety of exogenous or endogenous cellular stresses, and then blocks cellular malignant transformation, inhibits tumor progression, or promotes sensitization to radio-/chemo-therapy by mainly inducing cell cycle arrest, cell apoptosis, and cell senescence [18]. As a well-characterized transcription factor, p53 plays a powerful anti-tumor role by coordinating the expression of a set of target genes, such as *CDKN1A* (encoding the protein P21 that mediates cell cycle arrest) and *BBC3* (encoding the protein PUMA that mediates cell apoptosis) [19]. It is worth noting that the *TP53* gene mutation is one of the most common genetic alterations in many types of human tumors, including NSCLC with a mutation frequency of approximately 70% [20]. Missense mutations attributable to single-base substitutions within exons 5–8 (DNA binding domain, DBD) are the most common type of mutations observed in the *TP53* gene, which usually result in partial or even complete loss of p53 functions [20]. More than that, many types of mutant p53 (mut-p53) have been widely reported to acquire novel tumor-promoting phenotypes opposed to the wild-type p53 (wt-p53) via transcription- or non-transcription-dependent mechanisms, termed “Gain-of-Function” (GOF) [21]. Of note is that the distribution of *TP53* mutations in human tumors is not completely scattered but rather presents “hotspot” mutations that are centralized at codons 175, 248, and 273 [20]. In reality, the above-mentioned hotspot mutations have been demonstrated to exhibit varying aspects of GOF activity in lung cancer, including accelerating tumorigenesis, facilitating tumor invasion and metastasis, and diminishing host survival [22]. Given the irreplaceable prominence of the *TP53* gene in tumor biology, tumor management or therapeutic strategies targeting p53 have been attempted for decades. To date, however, most of these endeavors have met with little success [18]. This demoralizing fact suggests that the regulatory mechanisms of the p53 signaling pathway may go far beyond the tip of the iceberg that has been uncovered by far. In addition, the GOF activity shared by different mut-p53 may also be driven by their own unique molecular pathways [21]. Thereupon, clarifying the GOF activity of each mut-p53 and its underlying impacts on lung cancer progression and prognosis in the context of a specific mutation site, may contribute to achieving a more precise monitoring and management of lung cancer.

In our current work, p53 status was found to be the critical molecular switch that controls the role reversal of FOSB in determining the fate of NSCLC cells. By use of a large NSCLC cohort from The Cancer Genome Atlas (TCGA) database, our data showed that in lung cancer patients carrying wild-type *TP53*, FOSB expression betokened a positive prognosis; whereas in those carrying mutant *TP53*, it correlated with a poor one. Subsequently, the “friend” or “foe” identity of FOSB in tumor biology was validated through the construction of a range of NSCLC cells expressing different statuses of p53. Finally, three specific genetic backgrounds of *TP53*, *TP53-Null*, *TP53-WT*, and *TP53-R248Q*, were screened out to respectively elucidate the underlying molecular mechanisms by which FOSB has pluralistic effects on lung cancer progression and prognosis in these indicated genetic contexts of *TP53*. Herein we pioneered a novel insight into the unique interactive mechanisms between the transcription factors FOSB and p53 that, p53 or its mutants may guide FOSB to recognize and bind to distinct promoter sequences by interplaying with FOSB in the nucleus, thereby regulating different signaling pathways or differentially affecting the same signaling pathway in a specific transcriptional target-dependent manner. Given our current findings, exact opposite strategies targeting FOSB might be potentially required in lung cancer populations harboring different genetic backgrounds of *TP53*.

## Methods

### Bioinformatics analysis

The mRNA expression profile, *TP53* mutation spectrum, and sample information of the non-small cell lung cancer (NSCLC) cohort used in this study were acquired from The Cancer Genome Atlas (TCGA) database (https://xena.ucsc.edu/) to analyze the differential expression of FOSB mRNA between NSCLC tissues and adjacent normal tissues, and the associations of its expression with clinicopathological characteristics, including tumor size and TNM stage, and prognostic parameters, including therapeutic response, tumor recurrence, and overall survival (OS), in NSCLC patients harboring different genetic backgrounds of *TP53*. The differential expression of FOSB protein between human bronchial epithelial cells and NSCLC cells was visualized via THE HUMAN PROTEIN ATLAS database (https://www.proteinatlas.org/) in the form of immunohistochemical staining. The molecular docking between FOSB (UniProt ID: P53539) and p53 (UniProt ID: P04637) was carried out via the HDOCK SERVER (http://hdock.phys.hust.edu.cn/) [23], of which the results were visualized by the PyMOL software (Version 3.0), a user-sponsored molecular visualization system on an open-source foundation, maintained and distributed by Schrödinger (https://www.pymol.org/). The potential binding sites of the transcription factor FOSB within the promoters (A DNA sequence spanning the upstream 2000 bp to the downstream 200 bp of the transcriptional start site) of *PREX1*, *IGFBP5*, *AKR1C3*, and *ALDH3A1* were predicted via the JASPAR transcription factor binding profile database (https://jaspar.elixir.no/).

### Clinical sample analysis

The research involving the participation of human subjects was approved by the Institutional Review Board of China Medical University (Shenyang, China) and was in strict compliance with the Declaration of Helsinki. A total of 63 patients pathologically diagnosed with primary NSCLC (33 cases of lung adenocarcinoma (LUAD) and 30 cases of lung squamous cell carcinoma (LUSC)) by at least two experienced oncologists at the Department of Thoracic Surgery in The First Hospital of China Medical University (Shenyang, China) were recruited into the present study. Upon the completion of the prior informed consent procedure, fresh tumor tissues and the adjacent normal tissues were collected intraoperatively and promptly transferred into the liquid nitrogen for preservation. Meanwhile, the demographic and clinicopathological data of all the research subjects included were documented in detail for follow-up analysis.

### Reverse transcription and real-time quantitative PCR (RT-qPCR)

For RNA isolation, total RNA was isolated from collected tissues or cells with the RNAiso Plus kit (Takara, Japan), of which the quality was validated by the values of A260/A280 and A260/A230 between 1.8 and 2.2. For RT-qPCR, 1 µg of total RNA was subjected to reverse transcription to obtain the cDNA with the PrimeScript™ RT reagent Kit with gDNA Eraser (Takara, Japan). Subsequently, 2 µL of cDNA was mixed with 10 µL of TB Green^®^ Premix Ex Taq™ II (Takara, Japan) and 1.6 µL of specific primer pairs (Sangon Biotech, China) to be amplified in the LightCycler^®^ 480 Instrument II (Roche, Switzerland) with the amplification procedure that mainly included 40 cycles of the denaturation at 95 ℃ for 5 s and the annealing/extension at 60 ℃ for 30 s. The primer pairs used for the RT-qPCR are shown in supplementary Table S1. Finally, the method of 2^−∆∆CT^ was applied to the relative quantification of mRNA expression levels, which were uniformly normalized to *GAPDH*.

### Western Blot

For protein extraction, total protein was extracted from collected tissues or cells on ice with the RIPA Lysis Buffer kit (Beyotime Biotech, China), of which the concentrations were measured and adjusted for consistency with the BCA Protein Assay kit (Takara, Japan) as per the manufacturer’s instructions. For Western Blot, 20 µg of total protein was separated via electrophoresis in the BeyoGel™ SDS-PAGE Precast Gel (Beyotime Biotech, China), and then transferred to the Immobilon^®^-FL PVDF Membrane (Millipore, Germany). Subsequently, the protein-bound membranes were in turn blocked with the QuickBlock™ Blocking Buffer (Beyotime Biotech, China) at room temperature for 15 min, incubated with primary antibodies against the proteins of interest at 4 ℃ overnight, and incubated with secondary antibodies conjugated with horseradish peroxidase (HRP) at room temperature for 60 min. Finally, the immunoreactive bands of the target proteins were visualized with the Chemistar™ High-sig Western Blotting Substrate (Tanon, China) and then measured by the ImageJ software (National Institutes of Health, the United States of America (USA), https://imagej.nih.gov/ij/) to quantify protein expression levels, which were ultimately normalized to GAPDH. The antibodies used for the Western Blot were as follows: FosB (5G4) Rabbit mAb (Cell Signaling Technology, USA, #2251, 1:1000), P53 Polyclonal antibody (Proteintech, USA, Cat No. 10442-1-AP, 1:5000), P21 Polyclonal antibody (Proteintech, USA, Cat No. 10355-1-AP, 1:1000), PUMA Polyclonal antibody (Proteintech, USA, Cat No. 55120-1-AP, 1:1000), Phospho-P53 (Ser 15) Polyclonal antibody (Proteintech, USA, Cat No. 28961-1-AP, 1:1000), PREX1 Polyclonal Antibody (Invitrogen, USA, #PA5-104004, 1:1000), Rac1 Polyclonal antibody (Proteintech, USA, Cat No. 24072-1-AP, 1:1000), IGFBP5 Polyclonal antibody (Proteintech, USA, Cat No. 55205-1-AP, 1:500), ALDH3A1 Polyclonal antibody (Proteintech, USA, Cat No. 15578-1-AP, 1:20000), AKR1C3 Polyclonal antibody (Proteintech, USA, Cat No. 11194-1-AP, 1:1000), p44/42 MAPK (Erk1/2) (137F5) Rabbit mAb (Cell Signaling Technology, USA, #4695, 1:1000), Phospho-p44/42 MAPK (Erk1/2) (Thr202/Tyr204) Antibody (Cell Signaling Technology, USA, #9101, 1:1000), Akt (pan) (C67E7) Rabbit mAb (Cell Signaling Technology, USA, #4691, 1:1000), Phospho-Akt (Ser 473) Antibody (Cell Signaling Technology, USA, #9271, 1:1000), Anti-Ki67 antibody [EPR3610] (Abcam, the United Kingdom, ab92742, 1:5000), GAPDH Polyclonal antibody (Proteintech, USA, Cat No. 10494-1-AP, 1:10000), Histone H3 Polyclonal antibody (Proteintech, USA, Cat No. 17168-1-AP, 1:8000), and HRP-conjugated Affinipure Goat Anti-Rabbit IgG (H + L) (Proteintech, USA, Cat No. SA00001-2, 1:10000).

### Detection of active RAC1 (RAC1-GTP)

The detection of active RAC1 was conducted with the Active Rac1 Pull-Down and Detection Kit (Thermo Fisher Scientific, USA) strictly following the manufacturer’s instructions. In brief, cells were lysed on ice with the Lysis/Binding/Wash Buffer for 5 min, followed by centrifugation at 16,000 × *g* at 4 ℃ for 15 min to obtain the supernatant (total lysate). Then, the cell lysate was incubated with a mixture of GST-human Pak1-PBD and glutathione resin at 4 ℃ for 1 h with gentle rocking. Next, the reaction mixture was washed with the Lysis/Binding/Wash Buffer three times, followed by centrifugation at 6000 × *g* for 15 s. Finally, the pulled-down active RAC1 was eluted from the glutathione resin with the reducing sample buffer composed of 1 part β-mercaptoethanol and 20 parts 2X SDS Sample Buffer, followed by centrifugation at 6000 × *g* for 2 min to acquire the eluted samples, which were then subjected to the Western Blot procedure as described above to detect the levels of active RAC1 (RAC1-GTP) after the thermal denaturation at 95 ℃ for 5 min. The main antibodies involved in this part were as follows: Anti-Rac1 Antibody (Thermo Fisher Scientific, USA, #16118, 1:1000) and Goat anti-Mouse IgG (H + L) Secondary Antibody, HRP (Invitrogen, USA, #31430, 1:20000).

### Cell culture

The human normal bronchial epithelial cell lines – 16HBE was generously gifted by Prof. Chen from Sun Yat-sen University [24]; HBE was purchased from the American Type Culture Collection (ATCC) (Virginia, USA); and BEAS-2B was purchased from the National Collection of Authenticated Cell Cultures (Shanghai, China). The human NSCLC cell lines – H1299, A549, H1650, PC-9, and H1975 were purchased from the National Collection of Authenticated Cell Cultures, and LK-2 was purchased from the Japanese Collection of Research Bioresources Cell Bank (Osaka, Japan). 16HBE and HBE cells were cultured with the Minimum Essential Medium (MEM) (Biological Industries, Israel); BEAS-2B, H1299, LK-2, and PC-9 cells were cultured with the Dulbecco’s Modified Eagle Medium (DMEM) (Biological Industries, Israel); A549 cells were cultured with the Dulbecco’s Modified Eagle Medium/Nutrient Mixture F-12 (DMEM/F12) (Biological Industries, Israel); and H1650 and H1975 cells were cultured with the Roswell Park Memorial Institute 1640 (RPMI 1640) (Biological Industries, Israel). Unless otherwise indicated, all culture mediums were supplemented with 10% of fetal bovine serum (Biological Industries, Israel) and 1% of penicillin-streptomycin (Sigma-Aldrich, USA). Throughout the research, the cells were maintained in a moist and sterile incubator (Thermo Fisher Scientific, USA) with 5% of CO_2_ at 37 ℃, and involved in experiments at the logarithmic growth phase.

### Lentivirus infection

In this study, lentivirus infection was introduced to obtain the NSCLC cells stably expressing different statuses of p53 using H1299 cells with endogenous p53 deficiency as the parent. The construction of p53-expressing lentiviral vectors was entrusted to OBiO Technology Corp., Ltd. (Shanghai, China). For lentivirus infection, cells were inoculated into a 6-well plate on the previous day, and after 24 h of incubation when the cell confluence reached approximately 30–40%, the purified viral supernatant containing polybrene (Sigma-Aldrich, USA) at a final concentration of 5 µg/mL was added to the cells for effective infection. After incubation for 72 h, the cells were treated with 2 µg/mL puromycin (Gibco™, USA) to screen for the clones that were successfully infected, which lasted for 2 weeks. Finally, the protein expression levels of p53 were detected by the Western Blot as described above to determine the efficiency of infection.

### Agarose gel electrophoresis and Sanger sequencing

Agarose gel electrophoresis was used to isolate and retrieve the PCR amplification products of the ectopically expressed p53, followed by Sanger sequencing to validate the accuracy of each *TP53* mutation site constructed. The total RNA extraction and reverse transcription were in turn performed as described above to obtain the cDNA, which was then mixed with the 2 × Taq Master Mix (Dye Plus) (Vazyme, China) and specific primer pairs (Sangon Biotech, China) to be amplified in the TaKaRa PCR Thermal Cycler Dice™ Gradient (Takara, Japan). The PCR amplification procedure mainly included the denaturation at 95 ℃ for 15 s, annealing at 60 ℃ for 15 s, and extension at 72 ℃ for 60 s, for a total of 30 cycles. The primer pairs used for the RT-PCR are shown in supplementary Table S2. For agarose gel electrophoresis, the PCR clones obtained were separated via electrophoresis in a 0.2% of agarose gel and then visualized by the Tanon 1600 Multifunctional Gel Image Analysis System (Tanon, China) under ultraviolet light. Finally, Sanger sequencing for the target PCR clones was entrusted to Sangon Biotech Co., Ltd. (Shanghai, China).

### Cell transfection

The FOSB-overexpressing plasmid (Accession number: NM_006732.3) was constructed by OBiO Technology Corp., Ltd. (Shanghai, China) using the pcDNA 3.1 vector. The small interfering RNAs (siRNAs) targeting *PREX1*, *IGFBP5*, *AKR1C3*, *ALDH3A1*, or *TP53* were designed and synthesized by GenePharma Biotech Co., Ltd. (Soochow, China). For cell transfection, cells were inoculated into a 6-well plate on the previous day, and 24-hour incubation later when the cell confluence reached 60–70%, the FOSB-overexpressing plasmid (P3000™ Reagent was additionally added for plasmid transfection) and the specific siRNAs were alone or co-transfected into cells using the Lipofectamine™ 3000 Reagent (Invitrogen, USA). Unless otherwise noted, tumor biological assays were performed after the transfection for 24 h, and the detection of molecular indices was carried out after that for 48 h. The sequences of the siRNA oligo involved are shown in supplementary Table S3.

### Cell counting Kit-8 (CCK-8) cell proliferation assay

The CCK-8 cell proliferation assay was used to assess the proliferative capability in different transfected NSCLC cells. In brief, cells were in turn digested, centrifuged, resuspended, and counted by an automated cell counter (Countstar, China). After adjusting the cell concentration, 100 µL of cell suspension containing 2000 cells was inoculated into a 96-well plate. Following 4, 24, 48, 72, 96, and 120 h of incubation, the old medium was removed and replaced with fresh medium containing 10% of CCK-8 reagent (Sigma-Aldrich, USA), and 2-hour incubation at 37 ℃ later, the optical density (OD) values in each group of cells were read by an automatic microplate reader (BioTek, USA) at the wavelength of 450 nm.

### Colony formation assay

The colony formation assay was used to evaluate the independent viability in different transfected NSCLC cells. In brief, cells were in turn digested, centrifuged, resuspended, and counted by the automated cell counter. After adjusting the cell concentration, 2 mL of cell suspension containing 300 cells was inoculated into a 6-well plate. Following 10–20 days of incubation at 37 ℃ when macroscopic clones (cell number > 50) were developed, the cells were fixed with methanol for 15 min and then stained with 0.5% of crystal violet for 30 min. Finally, the positive clones in each group were counted with the ImageJ software.

### Transwell migration/invasion assays

The Transwell migration/invasion assays were used to assess the migration and invasion capabilities in different transfected NSCLC cells. In brief, cells were in turn digested, centrifuged, resuspended in the serum-free medium, and counted by the automated cell counter. After adjusting the cell concentration, 100 µL of cell suspension containing 3–5 × 10^4^ (For migration) or 5–7 × 10^4^ (For invasion) cells was inoculated into the Transwell chambers (BIOFIL, China) coated (For invasion) or not (For migration) with the Matrigel^®^ matrix (Corning, USA), which were then placed into a 24-well plate containing 700 µL of complete medium. Following 24 h of incubation at 37 ℃, the cells were fixed with methanol for 15 min and then stained with 0.5% of crystal violet for 30 min. Finally, the cells crossing the basal membrane of the Transwell chamber were counted with the ImageJ software.

### CCK-8 cytotoxicity assay

The CCK-8 cytotoxicity assay was used to estimate the cisplatin sensitivity in different transfected NSCLC cells. In brief, cells were in turn digested, centrifuged, resuspended, and counted by the automated cell counter. After adjusting the cell concentration, 100 µL of cell suspension containing 3000–5000 cells was inoculated into a 96-well plate. Following 24 h of incubation at 37 ℃, the cells were treated with cisplatin (CAS: 15663–27–1, Sigma-Aldrich, USA) at the concentrations of 0, 1, 2, 4, 8, and 16 µg/mL for another 48 h. Next, the CCK-8 test procedure was implemented as described above. Finally, the relative cell viability in each group was calculated as per the following formula:

(OD_treatment_ - OD_blank_ / OD_control_ - OD_blank_) × 100%.

### Detection of apoptosis by flow cytometry

Flow cytometry was used to detect the apoptotic levels induced by cisplatin in different transfected NSCLC cells. In brief, cells were inoculated into a 6-well plate on the previous day, and 24-hour incubation later when the cell confluence reached 50–60%, the cells were treated with 2 µg/mL cisplatin for another 48 h. Next, the cells were collected and then incubated with the Annexin V-FITC and Propidium Iodide (PI) supplied by the Annexin V-FITC/PI Apoptosis Detection Kit (KeyGEN BioTECH, China) at room temperature, away from light, for 10 min. Finally, the prepared cell samples were sent into the BD FACSCanto™ II Flow Cytometer (BD Biosciences, USA) to visualize the apoptosis rate in each group of cells.

### Transcriptome sequencing and analysis

Transcriptome sequencing for the FOSB-overexpressing H1299 cells with the genetic backgrounds of *TP53-Null*, *TP53-WT*, or *TP53-R248Q* was entrusted to LC-Bio Technology Co., Ltd. (Hangchow, China). The volcano plots were used to visualize the differentially expressed genes (DEGs) (FC > 1.5, *P* < 0.05) in the FOSB-overexpressing H1299 cells with the genetic backgrounds of *TP53-Null*, *TP53-WT*, and *TP53-R248Q*. The Venn diagram was used to visualize the intersection sets of the up-regulated DEGs (*P* < 0.05) among the FOSB-overexpressing H1299 cells with the genetic backgrounds of *TP53-Null*, *TP53-WT*, and *TP53-R248Q*. The Gene Ontology (GO) enrichment analyses were respectively performed for the up-regulated DEGs (*P* < 0.05) in the FOSB-overexpressing H1299 cells with the genetic backgrounds of *TP53-Null*, *TP53-WT*, and *TP53-R248Q*, of which the results were visualized by bubble charts (https://geneontology.org/).

### Cell line-derived xenograft (CDX) model

The CDX model was used to monitor the tumorigenic capability in vivo of the different transfected NSCLC cells. The research involving the use of animal subjects was approved by the Animal Welfare and Ethics Committee of China Medical University (Shenyang, China). A total of sixty 6~8-week-old male BALB/c nude mice purchased from Beijing Vital River Laboratory Animal Technology Co., Ltd. (Beijing, China) were engaged in the present study. The animals were housed in a SPF laboratory animal room (3 mice per cage) with a 12-hour light-dark alternation, a humidity of 50–60% and a room temperature of 24 ± 1 ℃. Maintenance feed and filtered water were administered ad libitum throughout the whole experiment. For the establishment of the CDX model, 100 µL of cell suspension containing 1 × 10^6^ cells was inoculated subcutaneously into the armpit of each mouse. Following 4 weeks of dynamic monitoring, when the visible development of the xenografts was observed in all mice, the euthanasia procedure was carried out to isolate the subcutaneous tumor tissues. Finally, the collected xenografts developed by each group of cells were weighed with an electronic balance (Mettler-Toledo, USA) and then transferred to an ultra-low temperature freezer (Thermo Fisher Scientific, USA) at -80 ℃ for preservation.

### Co-immunoprecipitation (Co-IP) assay for nuclear complex

The detection of the protein-protein interaction between FOSB and p53 in the nucleus was performed with the Nuclear Complex Co-IP Kit (Active Motif, USA) in strict accordance with the manufacturer’s instructions. In brief, cells were collected with ice-cold PBS/Phosphatase Inhibitors, centrifuged at 430 × *g* for 5 min, and then resuspended in 1× Hypotonic Buffer. After 15 min of incubation on ice, the cell suspension was centrifuged at 14,000 × *g* for 30 s to obtain the pellet (nuclear fraction). Next, the nuclear pellet was incubated with a mixture of the Complete Digestion Buffer and the Enzymatic Shearing Cocktail at 4 ℃ for 90 min, followed by centrifugation at 14,000 × *g* for 10 min to obtain the supernatant (nuclear lysate). Subsequently, the collected nuclear complex was incubated with the specific antibodies against FOSB (Cell Signaling Technology, USA, #2251, 1:50) or p53 (Proteintech, USA, Cat No. 10442-1-AP, 1:150) at 4 ℃ overnight on a rotator to induce the formation of an immune complex, which was then in turn incubated with the Protein G magnetic beads at 4 ℃ for 1 h with rotation and eluted from the magnetic beads with ice-cold IP Wash Buffer to acquire purified protein samples. Finally, the Western Blot was conducted as described above to visualize the potential protein-protein interaction between FOSB and p53.

### Chromatin immunoprecipitation (ChIP)-qPCR assay

The ChIP assay was performed with the EZ-Magna ChIP™ A/G Chromatin Immunoprecipitation Kit (Merck Millipore, USA) as per the manufacturer’s instructions. In brief, cells were in turn crosslinked with 1% of formaldehyde for 10 min, sufficiently broken by sonication to yield DNA fragments between 200 and 1000 bp in length, and centrifuged at 10,000 × *g* for 10 min to obtain the supernatant (total lysate). Then, the cell lysate was incubated with a mixture of the specific antibody against FOSB (Cell Signaling Technology, USA, #2251, 1:50) and the Protein A/G magnetic beads at 4 ℃ overnight on a rotator to induce the development of a Protein A/G bead-antibody/chromatin complex, in which the DNA fragments of interest were subsequently eluted from the magnetic beads with the ChIP Elution Buffer containing Proteinase K. Following the purification process, the collected DNA fragments bound to FOSB were ultimately subjected to the qPCR assay as described above to visualize the potential interplays between FOSB and the promoters of *PREX1*, *IGFBP5*, *AKR1C3*, and *ALDH3A1*. The primer pairs used for the ChIP-qPCR are shown in supplementary Table S4.

### Statistical processing

Experimental data were characterized by mean$\:\:\pm\:\:$standard deviation ($\:\overline{X}\pm\:SD$), and processed with the GraphPad Prism Software (Version 10.1.1, GraphPad Software, USA). For clinical samples, the paired-samples t-test was used to evaluate the differential expression of the indicated mRNA and protein. For the NSCLC cohort from the TCGA database, the Log-rank survival analysis was used to assess the differences in overall survival (OS) between two groups. For others, the two-tailed Student’s t-test was used to estimate the differences between two groups, whereas the One-way analysis of variance (One-way ANOVA) was used to estimate the differences among multiple groups (≥ 3), followed by the Bonferroni’s correction as the post hoc test for pairwise comparisons. Unless otherwise specified, all in vitro assays were independently repeated at least three times, and a *P*-value less than 0.05 was deemed statistically significant.

## Results

### FOSB expression predicted opposite prognoses between lung adenocarcinoma (LUAD) and lung squamous cell carcinoma (LUSC)

**Fig. 1:**
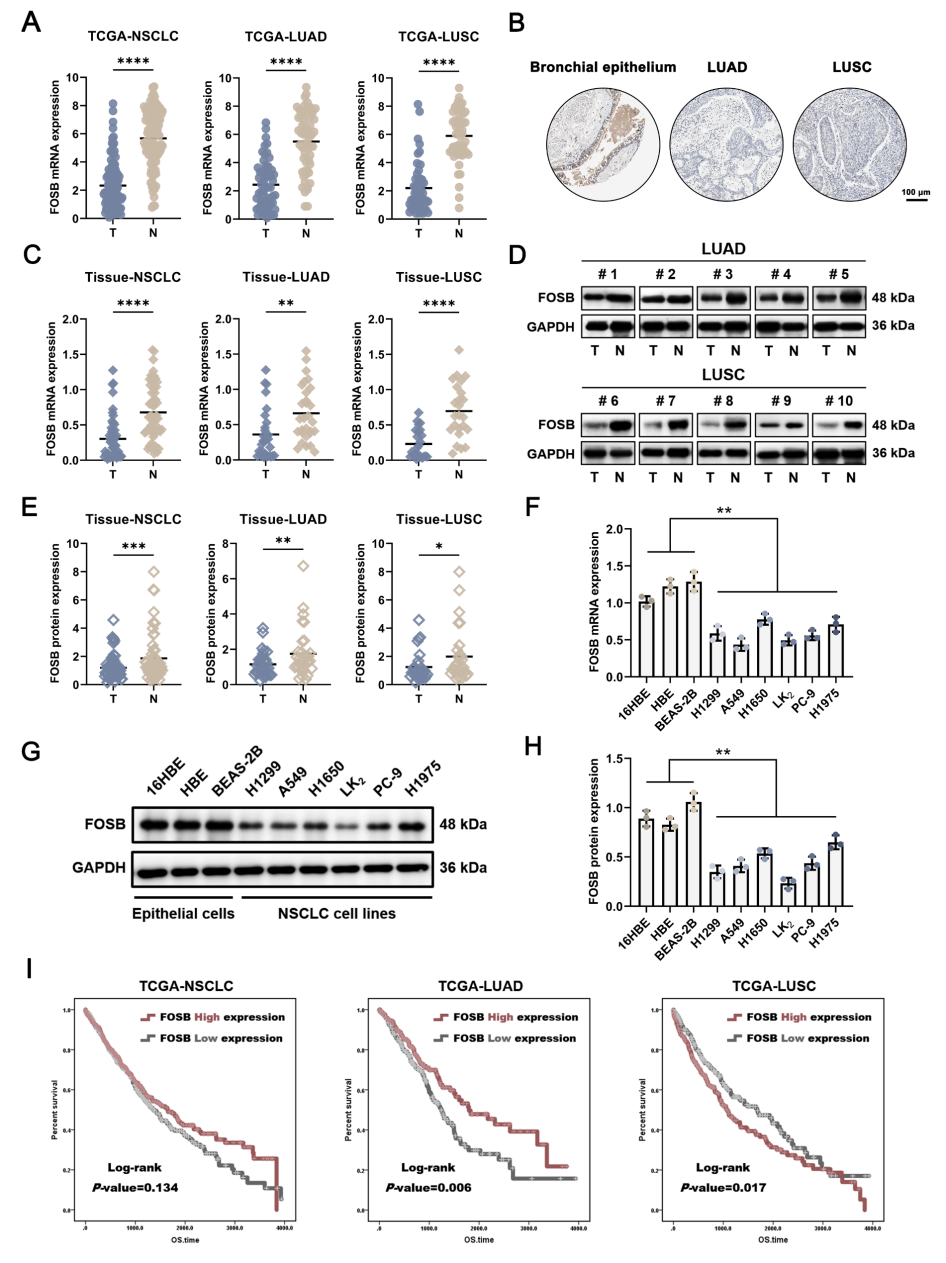
FOSB expression predicted opposite prognoses between LUAD and LUSC (**A**) FOSB mRNA expression profile in the NSCLC cohort, of which the data were obtained from the TCGA database; (**B**) FOSB protein expression levels in NSCLC tissues, of which the data were obtained from THE HUMAN PROTEIN ATLAS database; (**C**) FOSB mRNA expression levels in collected NSCLC tissues, detected by real-time quantitative PCR (RT-qPCR); (**D**) Representative immunoreactive bands of FOSB protein expression levels in collected NSCLC tissues, detected by Western Blot; (**E**) Quantitative analysis of the immunoreactive bands displayed in (D); (**F**) FOSB mRNA expression levels in NSCLC cell lines, detected by RT-qPCR; (**G**) Representative immunoreactive bands of FOSB protein expression levels in NSCLC cell lines, detected by Western Blot; (**H**) Quantitative analysis of the immunoreactive bands displayed in (G); (**I**) Kaplan-Meier survival curves (OS) of FOSB expression in the NSCLC cohort, of which the data were obtained from the TCGA database. * *P* < 0.05, ** *P* < 0.01, *** *P* < 0.001, **** *P* < 0.0001

To delineate the underlying clinical implications of FOSB expression in lung cancer, its molecular characteristics were revealed by use of a large non-small cell lung cancer (NSCLC) cohort from public databases and clinical NSCLC tissue samples. The Cancer Genome Atlas (TCGA) database showed that FOSB mRNA expression was significantly decreased in LUAD and LUSC compared to normal lung tissues (Fig. 1A). THE HUMAN PROTEIN ATLAS database indicated that FOSB protein was intensely positively expressed in bronchial epithelial cells while nearly negatively expressed in both LUAD and LUSC cells (Fig. 1B). Consistent with the observations in public databases, the markedly lower expression of FOSB mRNA (Fig. 1C) and protein (Fig. 1D-E) was also detected in collected NSCLC tissues than in paired paraneoplastic tissues. Additionally, the differential expression of FOSB between a group of human bronchial epithelial cell lines and NSCLC cell lines was further examined, and the results showed that FOSB mRNA (Fig. 1F) and protein (Fig. 1G-H) levels were consistently decreased in the NSCLC cell lines H1299, A549, H1650, LK_2_, PC-9, and H1975 compared to those in the bronchial epithelial cell lines 16HBE, HBE, and BEAS-2B. The above data suggested that the loss of FOSB expression might be involved in the malignant transformation of lung epithelial cells as a potential diagnostic biomarker for NSCLC. Considering the previously reported “two-sided” roles of FOSB in NSCLC, its prognostic effects on NSCLC patients were further evaluated via the Log-rank survival analyses. To our surprise, FOSB expression was tightly linked to diametrically opposite prognoses between LUAD and LUSC, that its expression correlated with a longer overall survival (OS) in LUAD, while a shorter one in LUSC (Fig. 1I). This striking finding demonstrated that the “two-sided” roles of FOSB in NSCLC might be traced to different histological subtypes.

### FOSB expression predicted opposite prognoses between NSCLC carrying wild-type and mutant *TP53*

**Fig. 2:**
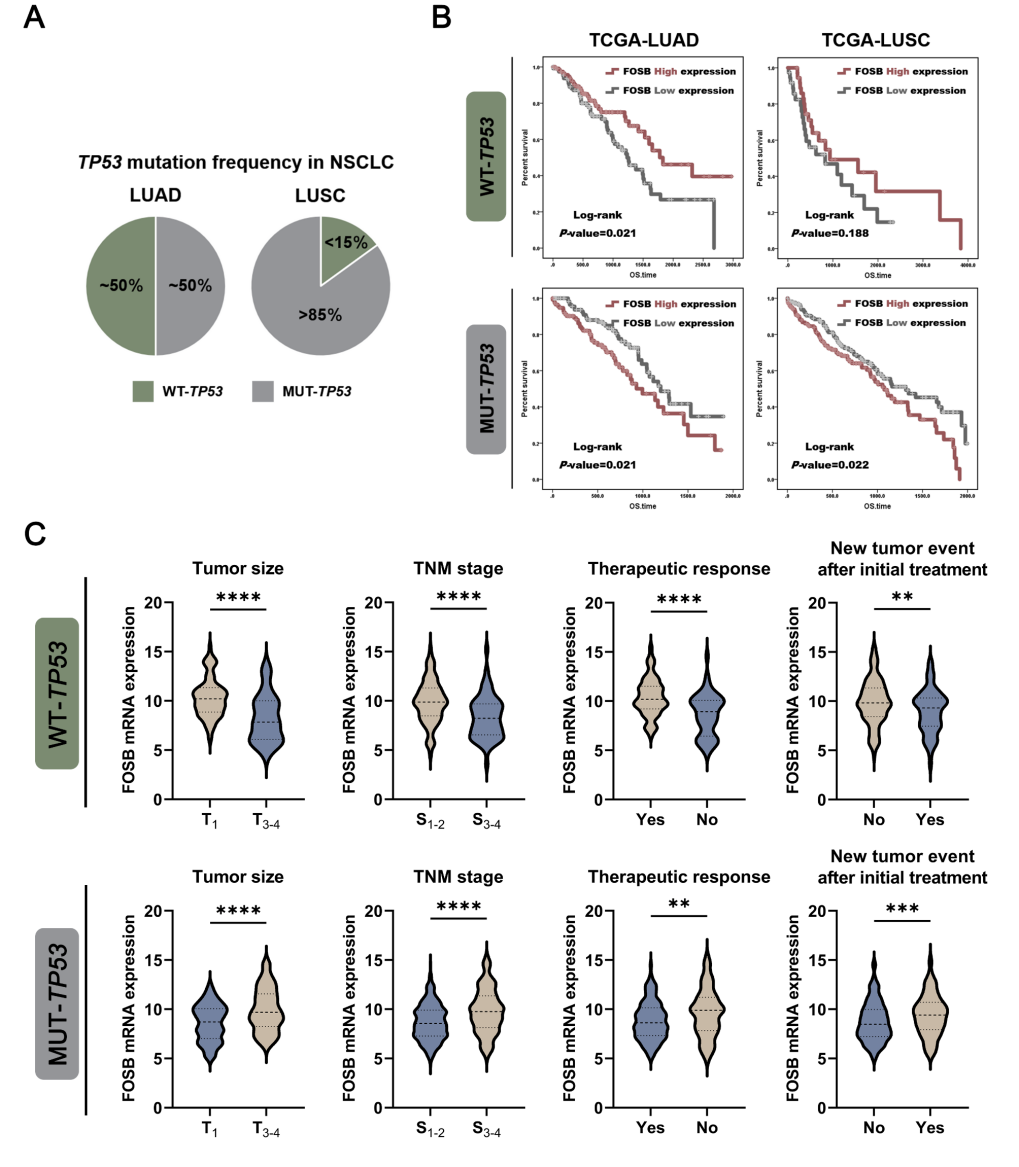
FOSB expression predicted opposite prognoses between NSCLC carrying wild-type and mutant *TP53* (**A**) The mutation frequency of the *TP53* gene in NSCLC, of which the data were obtained from the TCGA database; (**B**) Kaplan-Meier survival curves (OS) of FOSB expression in the NSCLC cohort bearing wild-type or mutant *TP53*, of which the data were obtained from the TCGA database; (**C**) Associations of FOSB expression with the clinicopathological characteristics and prognostic indices in the NSCLC cohort bearing wild-type or mutant *TP53*, of which the data were obtained from the TCGA database. ** *P* < 0.01, *** *P* < 0.001, **** *P* < 0.0001

Although the “dichotomy” system between LUAD and LUSC for NSCLC is principally based on histological and clinicopathological characteristics, emerging evidence highlights the remarkable heterogeneity in genetic and epigenetic maps, signal transduction networks, and immune landscapes between these two types of tumors [25, 26]. Therefore, we performed stratification analyses for the prognostic effects of FOSB expression based on a series of genetic molecular markers in both LUAD and LUSC as an attempt to identify the underlying critical determinants that contribute to the contradictory prognostic effects of FOSB in NSCLC (Data not shown).Notably, the *TP53* gene stood out for its remarkable disparity in the mutation frequency between LUAD and LUSC, which was approximately 50% in LUAD versus over 85% in LUSC (Fig. 2A). In light of the parallel relationship between the distinct antithesis in the tumor biological functions of wild-type (wt-) and mutant (mut-) p53 and the opposite prognostic effects of FOSB expression in LUAD and LUSC, we speculated that the mutation status of the *TP53* gene might serve as a golden key to unlocking the “two sides” of FOSB in NSCLC. As expected, the Log-rank survival analyses revealed that FOSB expression, in whether LUAD or LUSC, was significantly tied to a longer OS in patients harboring wild-type *TP53*, while a shorter one in those harboring mutant *TP53* (Fig. 2B). Furthermore, in the genetic context of wild-type *TP53*, FOSB expression was associated with a smaller tumor size, a lower TNM stage, a higher therapeutic response, and a more positive therapeutic outcome, suggesting potential anti-tumor effects of FOSB in NSCLC carrying wild-type *TP53* (Fig. 2C). In contrast, in the genetic context of mutant *TP53*, FOSB expression correlated with a larger tumor size, a higher TNM stage, a lower therapeutic response, and a more negative therapeutic outcome, implying potential pro-tumor effects of FOSB in NSCLC harboring mutant *TP53* (Fig. 2C). These data convincingly supported that the mutation status of the *TP53* gene might be a critical molecular switch that controls the diametrically opposite roles of FOSB in determining the fate of NSCLC.

### FOSB had heterogenous impacts on the tumor biology in NSCLC cells expressing p53 in variable statuses

**Fig. 3:**
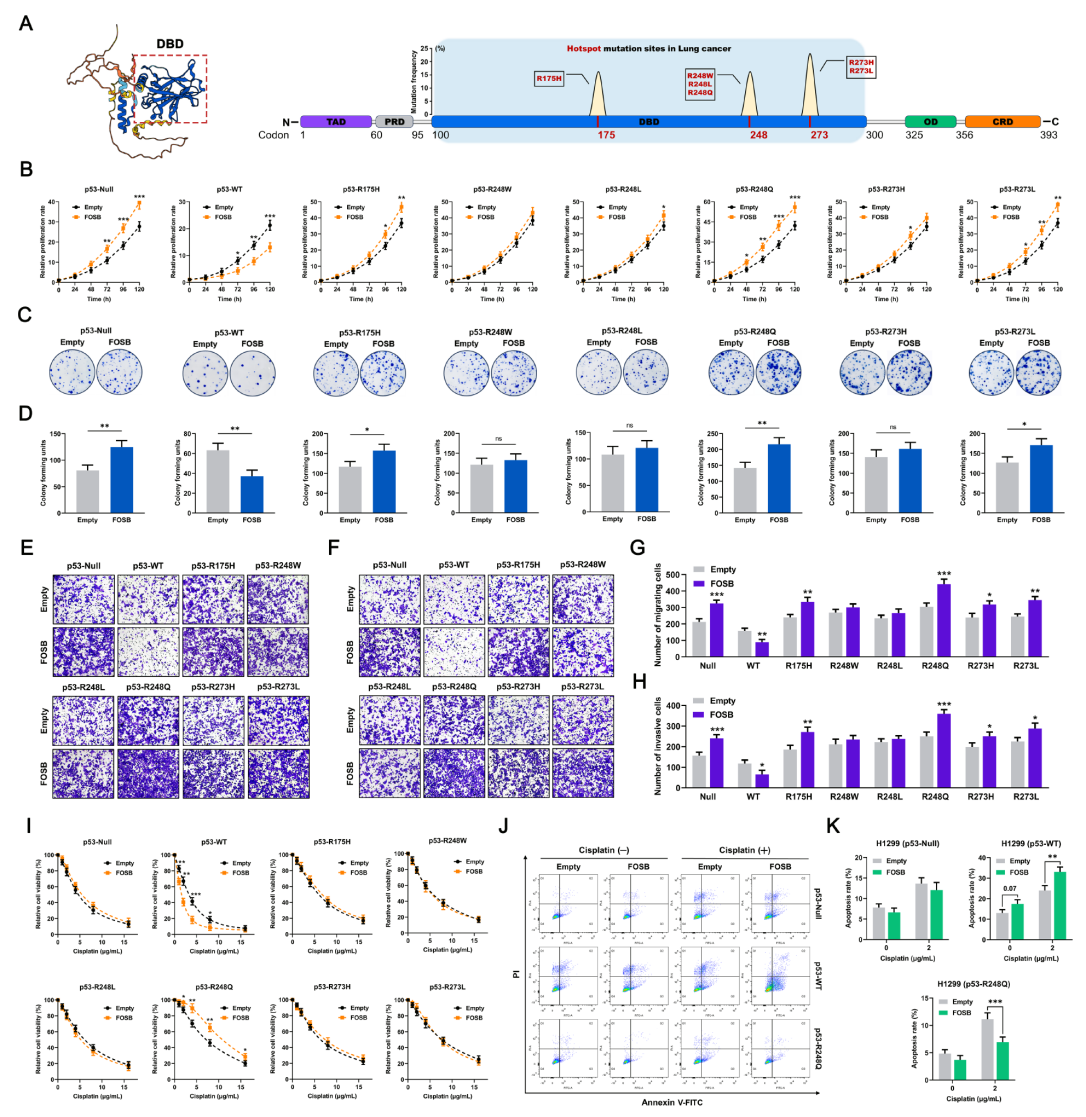
FOSB had heterogenous impacts on the tumor biology in NSCLC cells expressing p53 in variable statuses (**A**) The sites, frequency, and specific types of the “hotspot” mutations within the DBD of the *TP53* gene in NSCLC, of which the data were obtained from publications issued; (**B**) Effects of FOSB overexpression on the relative proliferation rate in H1299 cells expressing p53 in different statuses, evaluated by the CCK-8 cell proliferation assay; (**C**) Effects of FOSB overexpression on the independent viability in H1299 cells expressing p53 in different statuses, evaluated by the colony formation assay; (**D**) Quantitative analysis of the colony forming units displayed in (C); (**E**) Effects of FOSB overexpression on the migration capability in H1299 cells expressing p53 in different statuses, assessed by the Transwell migration assay; (**F**) Effects of FOSB overexpression on the invasion capability in H1299 cells expressing p53 in different statuses, assessed by the matrix gel-coated Transwell invasion assay; (**G**) Quantitative analysis of the number of migrating cells displayed in (E); (**H**) Quantitative analysis of the number of invasive cells displayed in (F); (**I**) Effects of FOSB overexpression on the cisplatin sensitivity in H1299 cells expressing p53 in different statuses, estimated by the CCK-8 cytotoxicity assay; (**J**) Effects of FOSB overexpression on the cisplatin-induced cell apoptosis in H1299 cells expressing p53 in different statuses, estimated by the flow cytometry; (**K**) Quantitative analysis of the apoptosis rate displayed in (J). * *P* < 0.05, ** *P* < 0.01, *** *P* < 0.001

*TP53* mutations are numerous in variety and may exhibit a dizzying array of Gain-of-Function (GOF) activity via comparatively independent molecular pathways. Thus, it is essential to validate, at the cellular level, and in the context of a specific *TP53* mutation site, the “two sides” of the tumor biological effects of FOSB observed in the NSCLC population with different genetic backgrounds of *TP53*. It has been reported that codons 175, 248, and 273 within the DNA binding domain (DBD) of the *TP53* gene are the “hotspot” mutation sites where missense mutations occur most frequently in human tumors including NSCLC [20, 27]. Among these missense mutations, p53-R175H, p53-R248W, p53-R248L, p53-R248Q, p53-R273H, and p53-R273L have been demonstrated to exert various aspects of GOF activity (Fig. 3A) [28–31]. Accordingly, we constructed a panel of syngeneically derived NSCLC cell models respectively expressing wild-type p53 (p53-WT) or the above site-specific mutant p53 using the NSCLC cell line H1299, which is deficient in endogenous p53 expression [28]. And then, the products of these ectopically expressed p53 were subjected to PCR amplification using specific primer pairs targeting codon 175 or a combination of codons 248 and 273, followed by validation of the accuracy of each mutation site via Sanger sequencing for the reclaimed PCR clones (Fig. S1A-B). In these H1299 cells ectopically expressing different statuses of p53, it was observed that the p53-WT significantly induced the expression of its specific targets P21 and PUMA, whereas all the p53 mutants tested almost entirely lost the ability to activate the p53 signaling pathway (Fig. S1C-D). In order to further investigate the potential effects of FOSB on the malignant biological behaviors in NSCLC cells under various genetic backgrounds of *TP53*, it was overexpressed in the above H1299 cells stably expressing p53 in different statuses (Fig. S2A-B). The CCK-8 cell proliferation assay showed that FOSB overexpression significantly promoted the proliferation in NSCLC cells expressing p53-Null, p53-R175H, p53-R248Q, and p53-R273L, while inhibited the proliferation in those expressing p53-WT (Fig. 3B). The colony formation assay indicated that FOSB overexpression greatly enhanced the independent viability in NSCLC cells expressing p53-Null, p53-R175H, p53-R248Q, and p53-R273L, while substantially impaired the independent viability in those expressing p53-WT (Fig. 3C-D). The Transwell assays revealed that FOSB overexpression imparted more active migratory (Fig. 3E, G) and invasive (Fig. 3F, H) properties to NSCLC cells expressing p53-Null, p53-R175H, p53-R248Q, p53-R273H, and p53-R273L, while deprived those expressing p53-WT of the potential for migration (Fig. 3E, G) and invasion (Fig. 3F, H). Taking into account the puzzling roles of FOSB in several common chemotherapeutic agents-induced cytotoxicity in NSCLC, the potential influences of FOSB expression on the cisplatin sensitivity in H1299 cells expressing p53 in different statuses were also evaluated in our current work. The CCK-8 cytotoxicity assay demonstrated that FOSB overexpression markedly increased the cisplatin sensitivity in NSCLC cells expressing p53-WT, while particularly reduced the cisplatin sensitivity in those expressing mutant p53-R248Q (Fig. 3I). Following these findings, the underlying impacts of FOSB expression on the cell apoptosis induced by cisplatin were further assessed in H1299 cells expressing p53-Null, p53-WT, and p53-R248Q. The data collected manifested that FOSB overexpression significantly exacerbated cisplatin-induced apoptosis in the genetic context of wild-type *TP53*, while obviously attenuated cisplatin-induced apoptosis in the genetic context of *TP53-R248Q* (Fig. 3J-K). Taken together, our current work confirmed the “two-sided” roles of FOSB in the malignant biological behaviors and cisplatin sensitivity in NSCLC cells with different genetic backgrounds of *TP53*. Since among all the mutation sites that were tested, FOSB overexpression exhibited the maximal facilitating effects on the malignant phenotypes (Fig. 3B-H) in NSCLC cells and a particular impact on their cisplatin sensitivity (Fig. 3I-K) in the presence of p53-R248Q, it was determined as the most meaningful and representative mutation site to be further investigated in follow-up studies. In addition, p53-Null, a special type of *TP53* mutations, was also brought into consideration in the subsequent mechanistic studies due to the lack of p53 protein expression.

### FOSB overexpression induced unique transcriptomic alterations in NSCLC cells expressing p53 in variable statuses

**Fig. 4:**
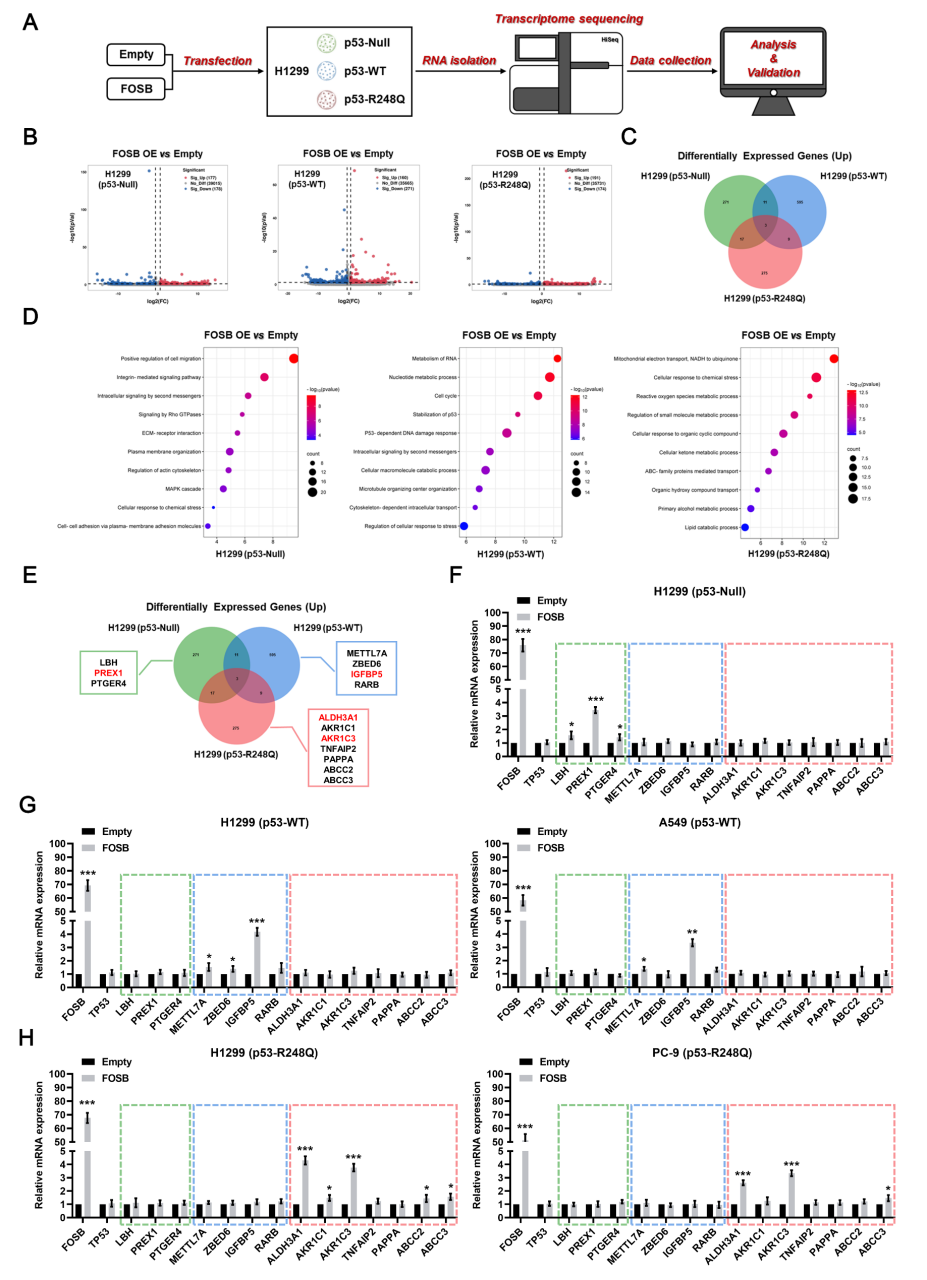
FOSB overexpression induced unique transcriptomic alterations in NSCLC cells expressing p53 in variable statuses (**A**) The schematic diagram illustrating the transcriptome sequencing process for H1299 cells expressing p53-Null, p53-WT, and p53-R248Q upon the transfection of the FOSB plasmid; (**B**) The volcano maps of the DEGs (FC > 1.5, *P* < 0.05) in H1299 cells expressing p53-Null, p53-WT, and p53-R248Q upon the transfection of the FOSB plasmid; (**C**) The Venn diagram showing the intersection sets among the three up-regulated DEGs sets (*P* < 0.05) in H1299 cells expressing p53-Null, p53-WT, and p53-R248Q upon the transfection of the FOSB plasmid; (**D**) The bubble diagrams presenting the top 10 biological processes significantly enriched by the GO enrichment analyses for the three up-regulated DEGs sets (*P* < 0.05) in H1299 cells expressing p53-Null, p53-WT, and p53-R248Q upon the transfection of the FOSB plasmid; (**E**) The Venn diagram exhibiting the candidate transcriptional targets of FOSB in H1299 cells expressing p53-Null, p53-WT, and p53-R248Q, respectively; (**F**) Effects of FOSB overexpression on the mRNA expression levels of its candidate transcriptional targets in H1299 cells expressing p53-Null, detected by the RT-qPCR; (**G**) Effects of FOSB overexpression on the mRNA expression levels of its candidate transcriptional targets in H1299 and A549 cells expressing p53-WT, detected by the RT-qPCR; (**H**) Effects of FOSB overexpression on the mRNA expression levels of its candidate transcriptional targets in H1299 and PC-9 cells expressing p53-R248Q, detected by the RT-qPCR. * *P* < 0.05, ** *P* < 0.01, *** *P* < 0.001

With an objective to explore the potential molecular mechanisms underlying the “two-sided” roles of FOSB in the malignant biological behaviors and cisplatin sensitivity in NSCLC cells expressing p53 in different statuses, three specific statuses of p53, namely the p53-Null, p53-WT, and p53-R248Q as highlighted above, were selected to be engaged in the following studies. Considering that FOSB is a well-characterized transcription factor that executes relevant biological functions mainly through transcriptional activation of the expression of specific target genes, the transcriptome sequencing in H1299 cells expressing p53-Null, p53-WT, or p53-R248Q was respectively performed upon the plasmid transfection-mediated FOSB overexpression, so as to identify its specific transcriptional targets over the assigned genetic backgrounds of *TP53* (Fig. 4A). The analysis of the differentially expressed genes (DEGs) showed that FOSB overexpression induced significant up-regulation (FC > 1.5, *P* < 0.05) of the expression of a total of 177, 160, and 191 genes in NSCLC cells expressing p53-Null, p53-WT, and p53-R248Q, respectively (Fig. 4B). Of particular interest, there was virtually no any shared subset among these three up-regulated DEGs sets, suggesting that FOSB overexpression elicited unique transcriptomic alterations in different genetic contexts of *TP53* specified above (Fig. 4C). To further determine the underlying biological processes significantly affected by FOSB overexpression under these specific genetic backgrounds of *TP53*, Gene Ontology (GO) enrichment analyses were conducted for the above three up-regulated DEGs sets, respectively. The results of the enrichment analyses revealed that the group of genes positively regulated by FOSB were closely involved in: (a) biological processes related to tumor promotion, including “Positive regulation of cell migration”, “Signaling by Rho GTPases”, and “MAPK cascade”, in NSCLC cells with p53 deficiency; (b) biological processes associated with tumor suppression, including “Cell cycle”, “Stabilization of p53”, and “P53-dependent DNA damage response”, in those expressing p53-WT; and (c) biological processes linked to cellular metabolic detoxication, including “Cellular response to chemical stress”, “Reactive oxygen species metabolic process”, and “Regulation of small molecule metabolic process”, in those expressing p53-R248Q (Fig. 4D). For the identification of the critical transcriptional targets by which FOSB regulated the above enriched biological processes, intersection sets were taken for the DEGs within the significantly enriched GO items, and 3 (*LBH*, *PREX1*, *PTGER4*), 4 (*METTL7A*, *ZBED6*, *IGFBP5*, *RARB*), and 7 (*ALDH3A1*, *AKR1C1*, *AKR1C3*, *TNFAIP2*, *PAPPA*, *ABCC2*, *ABCC3*) candidate transcriptional targets of FOSB were identified to be specific to *TP53-Null*, *TP53-WT*, and *TP53-R248Q*, respectively (Fig. 4E). Ultimately, through the validation by RT-qPCR, the Phosphatidylinositol-3,4,5-Trisphosphate Dependent Rac Exchange Factor 1 (*PREX1*) was identified as a specific transcriptional target of FOSB in H1299 cells with the genetic background of *TP53-Null* (Fig. 4F), the Insulin Like Growth Factor Binding Protein 5 (*IGFBP5*) was identified as a specific transcriptional target of FOSB in H1299 (Ectopically expressing p53-WT) and A549 (A NSCLC cell line expressing endogenous p53-WT [32]) cells with the genetic background of *TP53-WT* (Fig. 4G), and the Aldehyde Dehydrogenase 3 Family Member A1 (*ALDH3A1*) and Aldo-Keto Reductase Family 1 Member C3 (*AKR1C3*) were identified as two specific transcriptional targets of FOSB in H1299 (Ectopically expressing p53-R248Q) and PC-9 (A NSCLC cell line expressing endogenous p53-R248Q [33]) cells with the genetic background of *TP53-R248Q* (Fig. 4H). Consequently, we hypothesized that FOSB might induce unique changes in molecular events in NSCLC cells with a specific genetic background of *TP53* on the dependence of the above candidate transcriptional targets, respectively.

### FOSB differentially regulated downstream signaling pathways in a specific transcriptional target-dependent manner in NSCLC cells expressing p53 in variable statuses

**Fig. 5:**
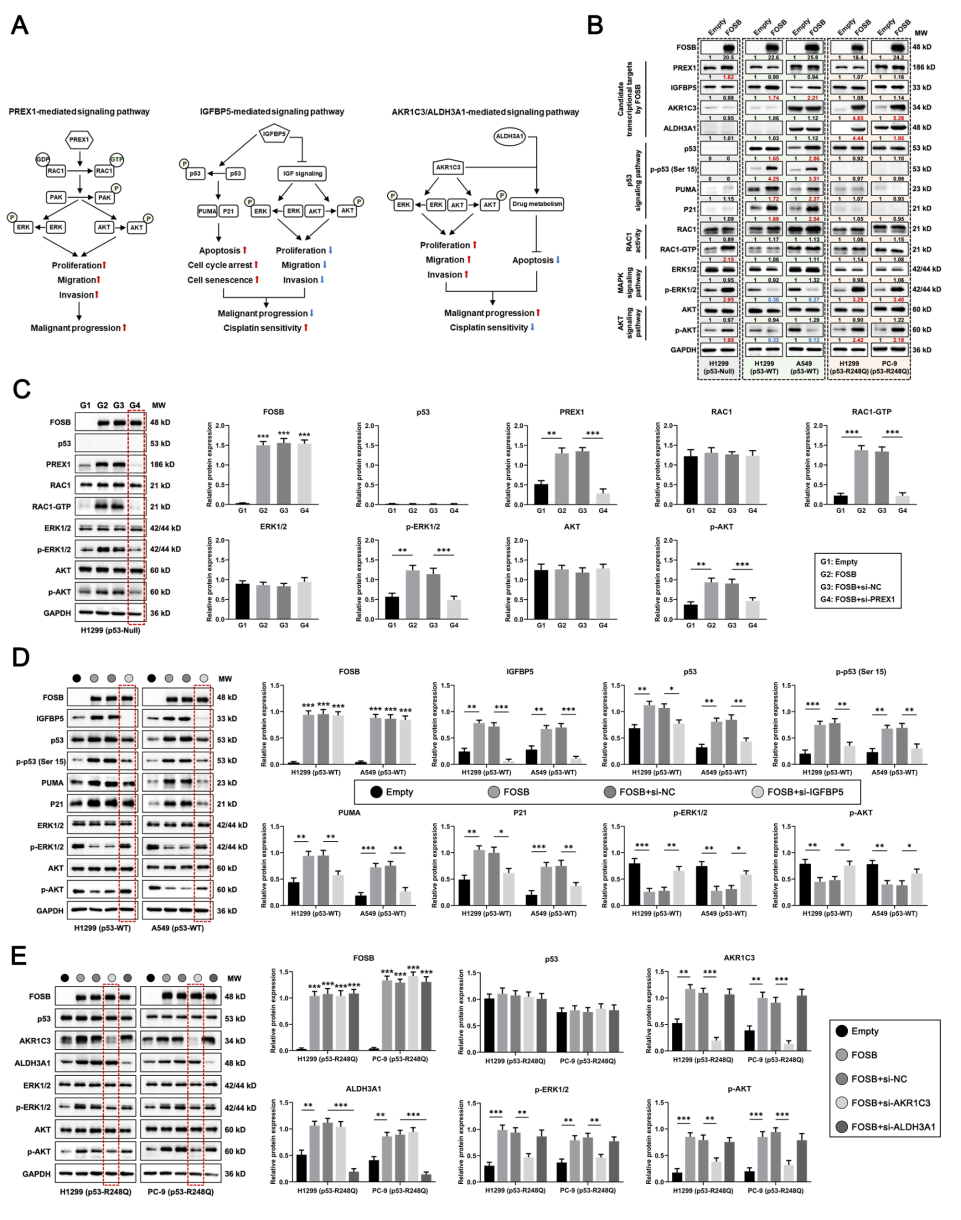
FOSB differentially regulated downstream signaling pathways in a specific transcriptional target-dependent manner in NSCLC cells expressing p53 in variable statuses (**A**) The abridged general view depicting the signaling pathways associated with tumor progression and prognosis that are respectively mediated by PREX1 (The left panel), IGFBP5 (The middle panel), AKR1C3 and ALDH3A1 (The right panel), summarized from the previous publications issued; (**B**) Effects of FOSB overexpression on the “PREX1-RAC1-MAPK (ERK)/AKT”, “IGFBP5-p53”, “IGFBP5-MAPK (ERK)/AKT”, and “AKR1C3-MAPK (ERK)/AKT” signaling pathways in H1299 (p53-Null), H1299 (p53-WT), A549 (p53-WT), H1299 (p53-R248Q), and PC-9 (p53-R248Q) cells, detected by the Western Blot; (**C**) Effects of the siRNA-mediated PREX1 knockdown on the regulation of the PREX1-RAC1-MAPK (ERK)/AKT oncogenic signaling pathways by FOSB in H1299 cells expressing p53-Null, detected by the Western Blot followed with quantitative analyses; (**D**) Effects of the siRNA-mediated IGFBP5 knockdown on the regulation of the IGFBP5-p53 oncosuppressive signaling pathway and the MAPK (ERK)/AKT oncogenic signaling pathways by FOSB in H1299 and A549 cells expressing p53-WT, detected by the Western Blot followed with quantitative analyses; (**E**) Effects of the siRNA-mediated AKR1C3 or ALDH3A1 knockdown on the regulation of the AKR1C3/ALDH3A1-MAPK (ERK)/AKT oncogenic signaling pathways by FOSB in H1299 and PC-9 cells expressing p53-R248Q, detected by the Western Blot followed with quantitative analyses. * *P* < 0.05, ** *P* < 0.01, *** *P* < 0.001

As demonstrated previously, the activation of the MAPK (The phosphorylation of ERK1/2) and AKT (The phosphorylation of AKT) oncogenic signaling pathways by PREX1 via the activation of its substrate RAC1 (The transformation of RAC1-GDP into RAC1-GTP) is intimately implicated in the malignant progression in human tumors [34] (Fig. 5A, the left panel); the activation of the p53 signaling pathway (The phosphorylation of p53 at ser 15) and the inhibition of the MAPK (The dephosphorylation of ERK1/2) and AKT (The dephosphorylation of AKT) oncogenic signaling pathways by IGFBP5 are associated with the tumor suppression and the platinum sensitization in human tumors [35, 36] (Fig. 5A, the middle panel); and the activation of the MAPK and AKT oncogenic signaling pathways by AKR1C3 [37] and the metabolic detoxification-induced chemo-resistance mediated by AKR1C3 and ALDH3A1 are major contributors to the tumor promotion and the tolerance to chemotherapy in human tumors [38, 39] (Fig. 5A, the right panel). Therefore, the potential effects of FOSB overexpression on the above signaling pathways in NSCLC cells with different genetic backgrounds of *TP53* were investigated by Western Blot analysis. The results showed that FOSB overexpression specifically gave rise to the activation of the PREX1-RAC1-MAPK (ERK)/AKT oncogenic signaling pathways in H1299 cells expressing p53-Null, rather than in the other NSCLC cells expressing p53-WT or p53-R248Q (Fig. 5B, lanes 1–2 from the left); FOSB overexpression specifically caused the activation of the IGFBP5-p53 tumor-suppressing signaling pathway and the inhibition of the MAPK (ERK)/AKT oncogenic signaling pathways in H1299 and A549 cells expressing p53-WT, rather than in the other NSCLC cells expressing p53-Null or p53-R248Q (Fig. 5B, lanes 3–6 from the left); and FOSB overexpression specifically elicited the activation of the AKR1C3/ALDH3A1-MAPK (ERK)/AKT oncogenic signaling pathways in H1299 and PC-9 cells expressing p53-R248Q, rather than in the other NSCLC cells expressing p53-Null or p53-WT (Fig. 5B, lanes 7–10 from the left). Next, it was inquired whether the regulation of the above signaling pathways by FOSB was predominantly reliant on the activation of its candidate transcriptional targets specific to a given genetic background of *TP53*. The data presented here evidently established that the activation of the PREX1-RAC1-MAPK (ERK)/AKT oncogenic signaling pathways by FOSB was significantly blocked by the small interfering RNA (siRNA)-mediated PREX1 knockdown in H1299 cells expressing p53-Null (Fig. 5C, lane 4 from the left); the activation of the IGFBP5-p53 oncosuppressive signaling pathway and the inhibition of the MAPK (ERK)/AKT oncogenic signaling pathways by FOSB were notably counteracted by the siRNA-mediated IGFBP5 knockdown in H1299 and A549 cells expressing p53-WT (Fig. 5D, lanes 4 and 8 from the left); and the activation of the MAPK (ERK)/AKT oncogenic signaling pathways by FOSB was markedly eliminated by the siRNA-mediated AKR1C3 (but not ALDH3A1) knockdown in H1299 and PC-9 cells expressing p53-R248Q (Fig. 5E, lanes 4 and 9 from the left). Overall, the activation of the above candidate transcriptional targets by FOSB, namely the *PREX1* (Specific for *TP53-Null*), *IGFBP5* (Specific for *TP53-WT*), and *AKR1C3* (Specific for *TP53-R248Q*), was confirmed to be indispensable for its unique regulation on the downstream oncogenic or oncosuppressive signaling pathways under a specific genetic background of *TP53*.

### FOSB had heterogenous impacts on the tumor biology in a specific transcriptional target-dependent manner in NSCLC cells expressing p53 in variable statuses

**Fig. 6:**
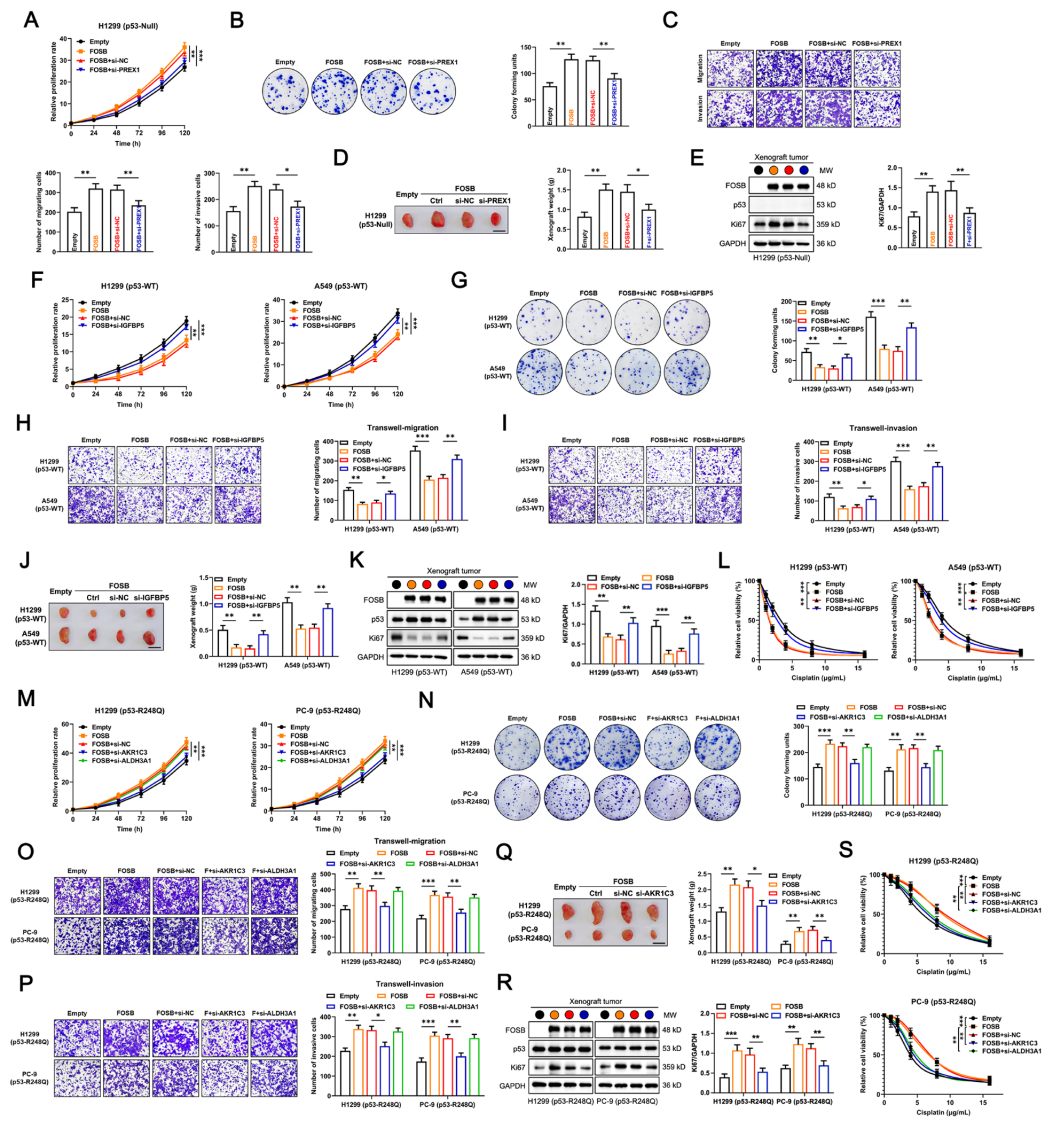
FOSB had heterogenous impacts on the tumor biology in a specific transcriptional target-dependent manner in NSCLC cells expressing p53 in variable statuses (**A**) Effects of PREX1 knockdown on the relative proliferation rate in H1299 (p53-Null) cells with FOSB overexpression, evaluated by the CCK-8 cell proliferation assay; (**B**) Effects of PREX1 knockdown on the independent viability in H1299 (p53-Null) cells with FOSB overexpression, evaluated by the colony formation assay; (**C**) Effects of PREX1 knockdown on the migration and invasion capabilities in H1299 (p53-Null) cells with FOSB overexpression, evaluated by the Transwell migration (Without the matrix gel coating) and invasion (With the matrix gel coating) assays; (**D**) Effects of PREX1 knockdown on the xenograft tumor growth in H1299 (p53-Null) cells with FOSB overexpression, evaluated by the cell line-derived xenograft (CDX) models (Scale bar: 1 cm, *n* = 3); (**E**) Effects of PREX1 knockdown on the protein expression levels of FOSB, p53, and Ki-67 in xenograft tumor tissues developed by H1299 (p53-Null) cells with FOSB overexpression, evaluated by the Western Blot with quantitative analysis; (**F**) Effects of IGFBP5 knockdown on the relative proliferation rate in H1299 (p53-WT) and A549 (p53-WT) cells with FOSB overexpression, assessed by the CCK-8 cell proliferation assay; (**G**) Effects of IGFBP5 knockdown on the independent viability in H1299 (p53-WT) and A549 (p53-WT) cells with FOSB overexpression, assessed by the colony formation assay; (**H**) Effects of IGFBP5 knockdown on the migration capability in H1299 (p53-WT) and A549 (p53-WT) cells with FOSB overexpression, assessed by the Transwell migration assay; (**I**) Effects of IGFBP5 knockdown on the invasion capability in H1299 (p53-WT) and A549 (p53-WT) cells with FOSB overexpression, assessed by the matrix gel-coated Tranwell invasion assay; (**J**) Effects of IGFBP5 knockdown on the xenograft tumor growth in H1299 (p53-WT) and A549 (p53-WT) cells with FOSB overexpression, assessed by the CDX models (Scale bar: 1 cm, *n* = 3); (**K**) Effects of IGFBP5 knockdown on the protein expression levels of FOSB, p53, and Ki-67 in xenograft tumor tissues developed by H1299 (p53-WT) and A549 (p53-WT) cells with FOSB overexpression, assessed by the Western Blot with quantitative analysis; (**L**) Effects of IGFBP5 knockdown on the cisplatin sensitivity in H1299 (p53-WT) and A549 (p53-WT) cells with FOSB overexpression, assessed by the CCK-8 cytotoxicity assay; (**M**) Effects of AKR1C3 or ALDH3A1 knockdown on the relative proliferation rate in H1299 (p53-R248Q) and PC-9 (p53-R248Q) cells with FOSB overexpression, estimated by the CCK-8 cell proliferation assay; (**N**) Effects of AKR1C3 or ALDH3A1 knockdown on the independent viability in H1299 (p53-R248Q) and PC-9 (p53-R248Q) cells with FOSB overexpression, estimated by the colony formation assay; (**O**) Effects of AKR1C3 or ALDH3A1 knockdown on the migration capability in H1299 (p53-R248Q) and PC-9 (p53-R248Q) cells with FOSB overexpression, estimated by the Transwell migration assay; (**P**) Effects of AKR1C3 or ALDH3A1 knockdown on the invasion capability in H1299 (p53-R248Q) and PC-9 (p53-R248Q) cells with FOSB overexpression, estimated by the matrix gel-coated Tranwell invasion assay; (**Q**) Effects of AKR1C3 knockdown on the xenograft tumor growth in H1299 (p53-R248Q) and PC-9 (p53-R248Q) cells with FOSB overexpression, estimated by the CDX models (Scale bar: 1 cm, *n* = 3); (**R**) Effects of AKR1C3 knockdown on the protein expression levels of FOSB, p53, and Ki-67 in xenograft tumor tissues developed by H1299 (p53-R248Q) and PC-9 (p53-R248Q) cells with FOSB overexpression, estimated by the Western Blot with quantitative analysis; (**S**) Effects of AKR1C3 or ALDH3A1 knockdown on the cisplatin sensitivity in H1299 (p53-R248Q) and PC-9 (p53-R248Q) cells with FOSB overexpression, estimated by the CCK-8 cytotoxicity assay. * *P* < 0.05, ** *P* < 0.01, *** *P* < 0.001

Subsequently, it was further verified whether the regulation of the above oncogenic or oncosuppressive signaling pathways by FOSB via its candidate transcriptional targets worked as the leading cause attributable to which it had heterogenous impacts on the malignant biological behaviors and cisplatin sensitivity in NSCLC cells carrying different genetic backgrounds of *TP53*. Firstly, in the genetic context of *TP53-Null*, the siRNA-mediated PREX1 knockdown was shown to distinctly interdict the promotion of proliferation (Fig. 6A), independent viability (Fig. 6B), migration, and invasion (Fig. 6C) by FOSB overexpression in NSCLC cells. Furthermore, PREX1 knockdown significantly reversed the promotion of growth in the xenograft tumors in vivo by FOSB overexpression in NSCLC cells (Fig. 6D), which was further validated by the decrease in Ki67 expression (Fig. 6E), a well-established molecular marker of cell proliferation [40]. Secondly, in the genetic context of *TP53-WT*, the siRNA-mediated IGFBP5 knockdown substantially restored the proliferation (Fig. 6F), independent viability (Fig. 6G), migration (Fig. 6H), invasion (Fig. 6I), as well as the xenograft tumor growth in vivo (Fig. 6J) and its associated Ki-67 expression (Fig. 6K) that were all suppressed by FOSB overexpression in NSCLC cells. Moreover, IGFBP5 knockdown severely deprived those expressing p53-WT of the increased sensitivity to cisplatin endowed by FOSB overexpression (Fig. 6L). Lastly, in the genetic context of *TP53-R248Q*, only the siRNA-mediated AKR1C3 knockdown was found to significantly abrogate the promotion of proliferation (Fig. 6M), independent viability (Fig. 6N), migration (Fig. 6O), invasion (Fig. 6P), together with the xenograft tumor growth in vivo (Fig. 6Q) and its associated Ki-67 expression (Fig. 6R) by FOSB overexpression in NSCLC cells. Notably, both the AKR1C3 and ALDH3A1 knockdown were demonstrated to considerably restore the sensitivity to cisplatin reduced by FOSB overexpression in those expressing p53-R248Q (Fig. 6S). Altogether, the above data substantiated the heterogenous impacts of FOSB on the malignant biological behaviors and cisplatin sensitivity in a specific transcriptional target-dependent manner in NSCLC cells bearing different genetic backgrounds of *TP53*.

### Wt- or mut-p53 guided FOSB to recognize and bind to distinct promoter sequences via protein-protein interactions to selectively transcriptionally activate specific target genes

**Fig. 7:**
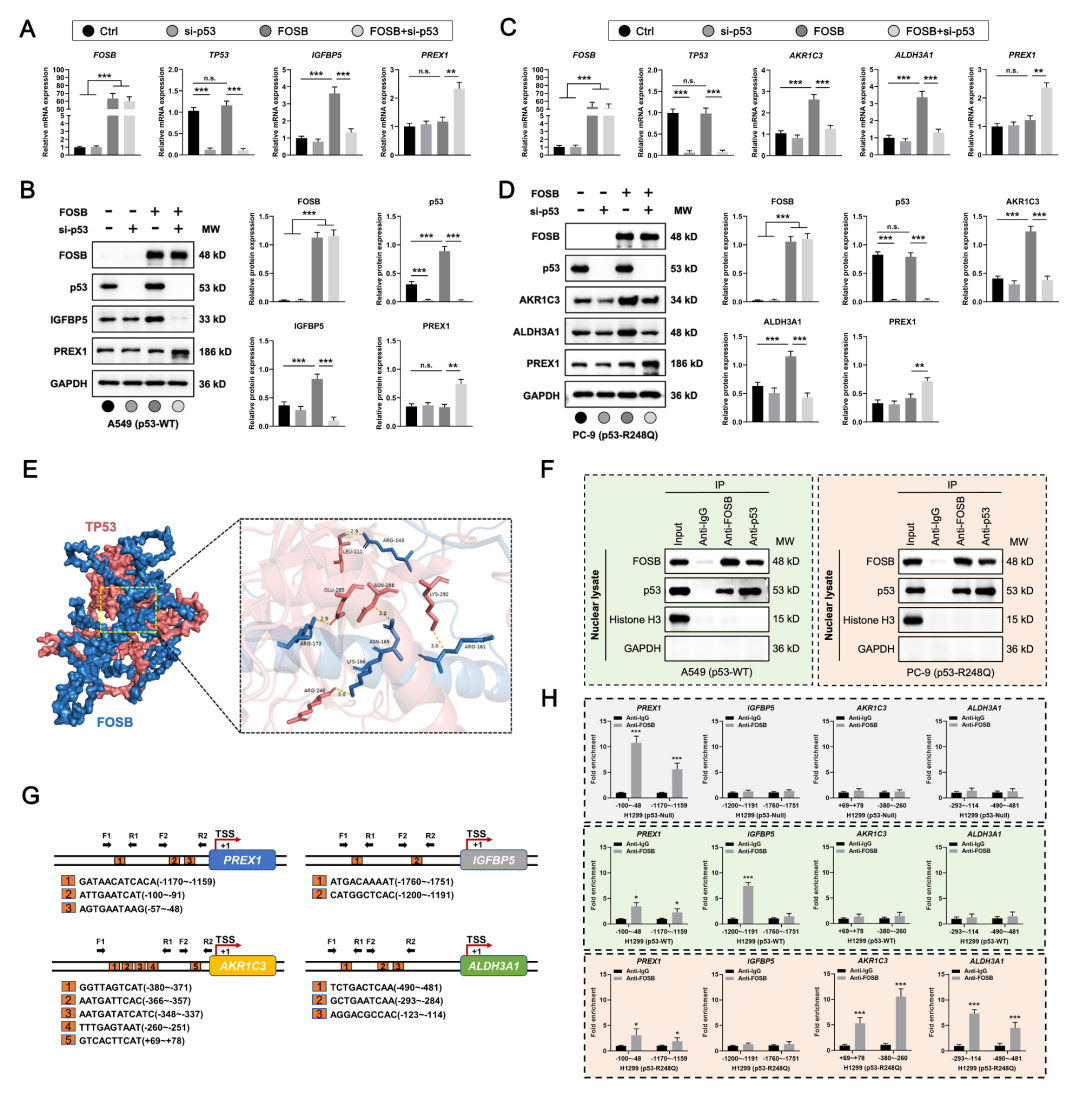
Wt- or mut-p53 guided FOSB to recognize and bind to distinct promoter sequences via protein-protein interactions to selectively transcriptionally activate specific target genes (**A**) Effects of p53 knockdown on the mRNA expression levels of *FOSB*, *TP53*, *IGFBP5*, and *PREX1* in A549 cells (p53-WT) with FOSB overexpression, detected by the RT-qPCR; (**B**) Effects of p53 knockdown on the protein expression levels of FOSB, p53, IGFBP5, and PREX1 in A549 cells (p53-WT) with FOSB overexpression, detected by the Western Blot followed with quantitative analyses; (**C**) Effects of p53 knockdown on the mRNA expression levels of *FOSB*, *TP53*, *AKR1C3*, *ALDH3A1*, and *PREX1* in PC-9 cells (p53-R248Q) with FOSB overexpression, detected by the RT-qPCR; (**D**) Effects of p53 knockdown on the protein expression levels of FOSB, p53, AKR1C3, ALDH3A1, and PREX1 in PC-9 cells (p53-R248Q) with FOSB overexpression, detected by the Western Blot followed with quantitative analyses; (**E**) The molecular docking model between FOSB and p53 exhibiting 5 pairs of interacting amino acid residues maintained by hydrogen bonds with the lengths between 2.9–3.1 Å, visualized by the PyMOL software; (**F**) The protein-protein interactions between FOSB and p53 (A549 cells), along with its mutant p53-R248Q (PC-9 cells) in the nucleus of NSCLC cells, detected by the nuclear complex co-immunoprecipitation combined with the Western Blot (Histone H3: indicator of nuclear fraction; GAPDH: indicator of cytoplasmic fraction); (**G**) The schematic diagram exhibiting the predicted binding sites of FOSB within the promoters of *PREX1*, *IGFBP5*, *AKR1C3*, and *ALDH3A1*, as well as the design strategy for the specific primer pairs used for the ChIP-qPCR, of which the data were obtained from the JASPAR database (F: Forward primer, R: Reverse primer, TSS: Transcriptional start site); (**H**) Effects of the ectopic expression of p53-WT or p53-R248Q on the selective binding of FOSB to the promoters of *PREX1*, *IGFBP5*, *AKR1C3*, and *ALDH3A1* in H1299 cells with p53 deficiency, detected by the ChIP-qPCR. * *P* < 0.05, ** *P* < 0.01, *** *P* < 0.001

Following the findings presented above, we proceeded to pursue the underlying molecular mechanisms by which FOSB selectively transcriptionally activated specific target genes under a certain genetic background of *TP53*. To begin with, it was observed whether the switch of p53 status was capable of triggering a corresponding shift in the candidate transcriptional targets selectively activated by FOSB in NSCLC cells. In A549 cells expressing endogenous p53-WT, FOSB overexpression specifically activated the expression of the transcriptional target *IGFBP5* at both the mRNA (Fig. 7A) and protein (Fig. 7B) levels, whereas the siRNA-mediated p53 knockdown (A switch in p53 status from p53-WT to p53-Null) led to a complete shift in the transcriptional targets of FOSB from *IGFBP5* towards *PREX1* (Fig. 7A-B). Likewise, in PC-9 cells expressing endogenous p53-R248Q, FOSB overexpression specifically activated the expression of the transcriptional targets *AKR1C3* and *ALDH3A1* at both the mRNA (Fig. 7C) and protein (Fig. 7D) levels, whereas the siRNA-mediated p53 knockdown (A switch in p53 status from p53-R248Q to p53-Null) resulted in a radical shift in the transcriptional targets of FOSB from *AKR1C3* and *ALDH3A1* towards *PREX1* (Fig. 7C-D). The above data highlighted the determinant role of p53 status in the selective activation of the candidate target genes by FOSB in NSCLC cells. In consideration of a well-established mechanism that wt- and mut-p53 may both interplay with an identical array of transcription factors to create entirely different impacts on the transcriptional events they mediate [41], it was consequently hypothesized that the p53 in diverse statuses might also interact with FOSB to reshape its selective preferences for transcriptional targets. To test this hypothesis, a molecular docking model between FOSB and p53 was established via bioinformatics analysis (Docking Score: -260.31, Confidence Score: 0.901), and a total of 90 pairs of interacting amino acid residues were predicted, among which 5 pairs (143 A–111 A, 161–292 A, 165–288 A, 166–248 A, and 173–285 A) maintained by hydrogen bonds with appropriate lengths (2.9–3.1 Å) were exhibited (Fig. 7E). More importantly, the protein-protein interactions between FOSB and p53, along with its mutant p53-R248Q in the nucleus, were definitely captured by nuclear complex co-immunoprecipitations with the specific antibodies against FOSB or p53 in A549 (p53-WT) and PC-9 (p53-R248Q) cells (Fig. 7F). Next, it was further investigated whether the interplays of p53 in varying statuses with FOSB would drive the changes in its selective binding to the promoters of the candidate target genes. To this end, the promoter analysis was performed, and a total of 3 (-1170~-1159, -100~-91, and − 57~-48), 2 (-1760~-1751 and − 1200~-1191), 5 (-380~-371, -366~-357, -348~-337, -260~-251, and + 69 ~ + 78), and 3 (-490~-481, -293~-284, and − 123~-114) potential binding sites of FOSB were respectively identified in the promoters of *PREX1*, *IGFBP5*, *AKR1C3*, and *ALDH3A1* by use of the JASPAR database, according to which specific primer pairs for the subsequent chromatin immunoprecipitation-qPCR (ChIP-qPCR) were designed and synthesized (Fig. 7G). The results of the ChIP-qPCR demonstrated that in H1299 cells with p53 deficiency, FOSB bound only to the promoter (-1170~-1159 and − 100~-48) of *PREX1* (Fig. 7H, the upper panel), whereas the ectopic expression of p53-WT substantially weakened the binding of FOSB to the *PREX1* promoter and significantly enhanced its binding to the promoter (-1200~-1191) of *IGFBP5* (Fig. 7H, the middle panel), and the ectopic expression of p53-R248Q considerably attenuated the binding of FOSB to the *PREX1* promoter and markedly increased its binding to the *AKR1C3* (-380~-251 and + 69 ~ + 78) and *ALDH3A1* (-490~-481 and − 293~-114) promoters (Fig. 7H, the lower panel). Taken together, these data collected here suggested that the wt- or mut-p53 might guide FOSB to recognize and bind to unique promoter sequences via well-coordinated protein-protein interactions to selectively transcriptionally activate specific target genes.

## Discussion

Endeavors towards the development of the Activator protein-1 (AP-1) transcription factor family as novel molecular biomarkers and therapeutic targets for human cancers have been ongoing due to their extensive involvement in all aspects of tumor biology and prominent clinical relevance as previously reported [8, 42]. However, it is worth noting that the complexity in the roles of AP-1 members and the regulation of their activity in tumor evolution may go far beyond our current knowledge: they are more likely to act as a flexible double-edged sword capable of driving tumor cells into entirely different fates, relying on specific histological types or genetic backgrounds [9, 43]. Bearing this in mind, it should come as no surprise that the FBJ Murine Osteosarcoma Viral Oncogene Homolog B (FOSB), a classical AP-1 transcription factor, has been reported to play two-sided roles in the progression and prognosis of non-small cell lung cancer (NSCLC) [11, 13, 16, 17]. Nevertheless, little has been known to date about the reasons for the two-sided properties of FOSB in NSCLC and the molecular mechanisms involved, which might emerge as a main barrier to the application of FOSB into the management tactics and clinical decision for NSCLC.

In our current work, an intriguing finding that FOSB expression indicated diametrically opposed prognoses between the two most common histological subtypes of NSCLC, lung adenocarcinoma (LUAD) and lung squamous cell carcinoma (LUSC), provided critical scientific clues for us to narrate this story that unraveled the mystery of its “two sides” in NSCLC (Fig. 1). The wide difference in the mutation frequency of the Tumor Protein P53 (*TP53*) gene between LUAD (~ 50%) and LUSC (> 85%) represented a reasonable hypothesis, that the opposite prognostic effects of FOSB expression between these two subtypes of NSCLC might highly depend on the genetic background of *TP53*, which was then validated by a large NSCLC cohort containing 331 samples with wild-type *TP53* and 648 samples with mutant *TP53* from The Cancer Genome Atlas (TCGA) database – FOSB expression predicted a longer overall survival (OS) and was associated with positive clinicopathological characteristics and responsiveness to therapy in patients harboring wild-type *TP53*, whereas the exact opposite was observed in those harboring mutant *TP53* (Fig. 2). By constructing a panel of syngeneically derived NSCLC cells expressing wild-type (wt-) p53 or various mutant (mut-) p53 that had been shown to be the “hotspot” mutations with Gain-of-Function (GOF) activity on the H1299 cells with p53 deficiency, our current work further validated the observations in the population study that, FOSB served as a tumor suppressor in NSCLC cells expressing wt-p53, while a tumor promoter in those expressing most types of the mut-p53 tested (including p53 deficiency as a special mutation type of *TP53*) (Fig. 3). Taken together, in combination with its underlying clinical implications and well-characterized tumor biological effects, our current work identified FOSB as a novel and promising prognostic biomarker for NSCLC with a given genetic background of *TP53*. Although rarely, to our knowledge, in addition to FOSB, there are other molecules whose tumor biological functions are also subjected to the determinative influences by p53 status. For instance, the overexpression of Centromere Protein A (CENP-A) was shown to promote cell cycle arrest and cell senescence, and increase the radiosensitivity in a p53-dependent manner in tumor cells bearing wild-type *TP53*, while instead drive tumor invasion and metastasis, and decrease its radiosensitivity upon the absence of functional p53 expression [44]. Furthermore, the blockade of the Neural Precursor Cell Expressed, Developmentally Down-Regulated 8 (NEDD8)-mediated ubiquitination-like modification Neddylation was demonstrated to have totally different impacts on the migration and invasion capabilities between tumor cells expressing wt- and mut-p53 [45]. Following these distinctive findings, we proposed herein a perspective that, the p53 (in varying statuses)-mediated intergenic interactions might contribute to pluralistic outcomes in tumor evolution by remodeling the roles of certain critical modulators of cell fate, which might represent a cluster of well-coordinated molecular biological events reaching far beyond the roles of p53 and its mutants themselves.

Despite the universal antithesis between the functions of wt- and mut-p53, a consensus has long been reached that different types of mut-p53 may exhibit a dizzying array of GOF activity via molecular pathways that are relatively independent of each other [21]. Therefore, it is absolutely required to investigate the molecular mechanisms underlying the transformation of the tumor biological functions of FOSB in the context of a specific *TP53* mutation site. Based on the well-validated tumor biological effects by FOSB overexpression in NSCLC cells across a range of different genetic backgrounds of *TP53*, three statuses of p53, namely the p53-Null, p53-WT, and p53-R248Q, were determined to be engaged in the follow-up mechanistic studies (Fig. 3). Subsequently, the transcriptome sequencing combined with the validation by RT-qPCR identified 4 candidate transcriptional targets of FOSB: *PREX1* (specific to *TP53-Null*), *IGFBP5* (specific to *TP53-WT*), *AKR1C3* and *ALDH3A1* (specific to *TP53-R248Q*) (Fig. 4). Rac Family Small GTPase 1 (RAC1), a well-defined oncoprotein in NSCLC, drives its malignant progression by enhancing the proliferation, migration, and invasion abilities in tumor cells, which may be implicated in the RAC1-mediated activation of the MAPK/ERK and PI3K/AKT oncogenic signaling pathways [34, 46]. As exuberant pro-survival and pro-proliferative signals, the constitutive activation of the MAPK/ERK and PI3K/AKT is frequently observed in most human tumors including NSCLC [47, 48]. The activation of RAC1 is of vital importance to the oncogenic signal transduction involved, in which the Phosphatidylinositol-3,4,5-Trisphosphate Dependent Rac Exchange Factor 1 (PREX1) has been extensively reported as a crucial modulator of its activity by converting the inactive RAC1-GDP into the active RAC1-GTP [34]. Thus, the regulation by FOSB on the RAC1-ERK/AKT oncogenic signaling pathways via the specific transcriptional target *PREX1* might be a critical molecular pathway through which it drives the malignant progression in NSCLC with the genetic background of *TP53-Null* (Figs. 5 and 6). The Insulin Like Growth Factor Binding Protein 5 (IGFBP5) is an important member of the IGFBP family that suppress the Insulin Like Growth Factor 1 Receptor (IGF1R)-mediated insulin-like growth signaling by competitively binding to the IGF1/2 against IGF1R, of which the MAPK/ERK and PI3K/AKT are the major effectors [49, 50]. As a potential tumor suppressor, IGFBP5 is capable of restraining tumor growth and progression via regulating the MAPK/ERK and PI3K/AKT signaling [36]. On the other hand, IGFBP5 was shown to induce the phosphorylation modification of p53 at ser 15 to enhance its stability and transcriptional activity, thereby executing anti-tumor functions in a p53-dependent manner [35]. Therefore, the transcriptional activation of *IGFBP5* by FOSB might work as a pivotal molecular pathway underlying its robust anti-tumor effects and promotion of the sensitization to cisplatin in NSCLC with the genetic background of *TP53-WT* (Figs. 5 and 6). Aldo-Keto Reductase Family 1 Member C3 (AKR1C3), a member of the AKR superfamily, was reported to endow human tumors with an array of malignant properties including overgrowth, invasion, metastasis, and drug resistance through multiple oncogenic signaling pathways [51]. Growing evidence reveals that both the MAPK/ERK and PI3K/AKT signaling cascades as the main effectors play a dominant role in AKR1C3-mediated malignant progression in tumors [52, 53]. Notably, the AKR1C3 and Aldehyde Dehydrogenase 3 Family Member A1 (ALDH3A1) are the two important metabolic enzymes in cells that have been well demonstrated to be equipped with tremendous potential for driving the development and maintenance of drug resistance in tumor cells by directly metabolizing chemotherapeutic agents or their derived cytotoxic products such as reactive oxygen species [38, 54]. Indeed, the use of AKR1C3 or ALDH3A1 inhibitors has been showing evident therapeutic benefits in preclinical research, highlighting their potential application value in the endeavor towards tumor treatment [55, 56]. Hence, the impacts of FOSB on the malignant biological behaviors and cisplatin sensitivity in tumor cells might be primarily attributable to the transcriptional activation of its specific transcriptional targets *AKR1C3* and *ALDH3A1* in NSCLC with the genetic background of *TP53-R248Q* (Figs. 5 and 6).

With the integration of bioinformatics analysis represented by the molecular docking and molecular biology approaches including the co-immunoprecipitation (Co-IP) and chromatin immunoprecipitation (ChIP), our current work revealed a novel and well-coordinated protein-protein interaction network between FOSB and p53, in which the wt- or mut-p53 may guide FOSB to recognize and bind to distinct promoter sequences to transcriptionally activate the expression of specific target genes (Fig. 7). As a matter of fact, it has been well established that the activity and functions of AP-1 are highly modulated by sophisticated and diversifying transcription factor complexes, in which an increasing number of interacting proteins are being continuously identified as their potential transcriptional co-activators [57, 58]. It has been recently reported that the KAT5 served as a transcriptional partner of FOSB to potentiate thyroid cancer growth and metastasis by enhancing FOSB-mediated transcriptional activation of *DPP4* [59]. Furthermore, the transcriptional activation of *MMP7* by the FOSB-MAFG transcription factor complex was demonstrated to be critical for the malignant progression in hepatocellular carcinoma [60]. However, the underlying interacting proteins of FOSB in NSCLC have rarely been characterized to date, despite the fact that they may have overwhelming impacts on FOSB-mediated transcriptional events that determine the fate of tumor cells. Of note, although p53 is also characterized as a transcription factor capable of independently regulating the expression of its own group of target genes, the interactions of p53 with other transcription factors have been shown to be another critical molecular pathways for the execution of its biological functions [61]. Regarding the mut-p53 that have lost the tumor suppressing capability, the GOF activity they may acquire is also found to be largely dependent on their interactions with a wide variety of transcription factors [62]. Interestingly enough, there is emerging evidence that p53 and its mutants may interplay with the same transcription factor to remodel its choice preference for candidate transcriptional targets [41, 63]. Herein, we identified the transcription factor FOSB as a novel cooperative partner shared by both p53 and its mutant p53-R248Q, which revealed a unique and well-coordinated intergenic interaction network between *FOSB* and *TP53* that may have profound impacts on the evolution of NSCLC.

## Conclusions

### Identification of FOSB expression as a novel prognostic biomarker for NSCLC, in combination with the mutation status of *TP53*

The prognostic effects of the transcription factor FOSB expression in NSCLC are dependent on the mutation status of the *TP53* gene: its expression indicates a positive prognosis in NSCLC patients harboring wild-type *TP53* while a negative one in those harboring mutant *TP53*. Accordingly, FOSB expression holds promise as a novel prognostic biomarker for NSCLC in combination with a specific genetic background of *TP53*.

### Identification of the interactions between FOSB and p53 as potential intervention targets for NSCLC

**Fig. 8:**
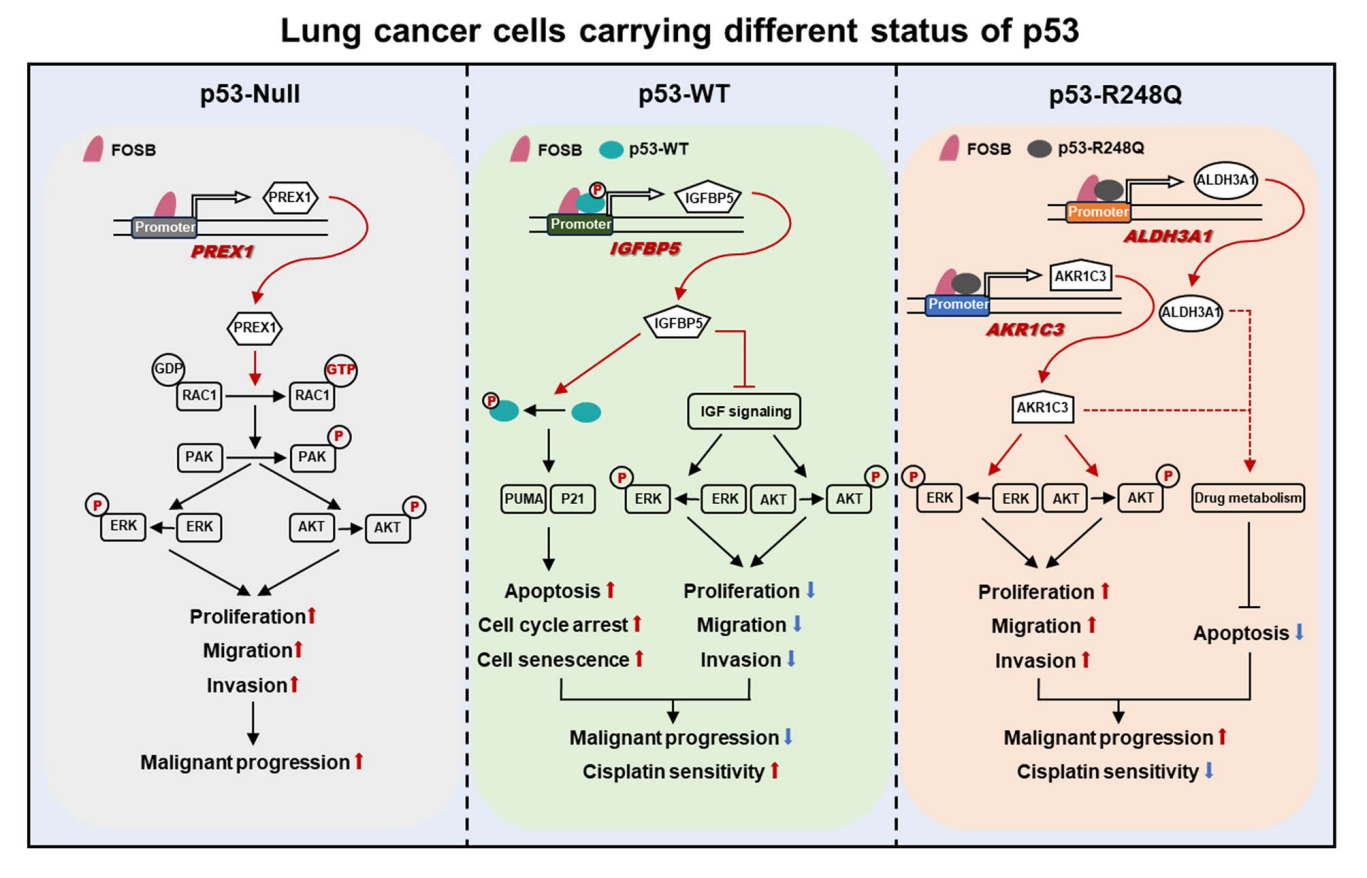
Two-polarized roles of transcription factor FOSB in lung cancer progression and prognosis on the dependence of p53 status

There is a unique and well-coordinated protein-protein interaction network between the transcription factors FOSB and p53, in which wt- or mut-p53 may guide FOSB to recognize and bind to distinct promoter sequences to transcriptionally activate the expression of specific target genes, thereby resulting in overwhelmingly opposite impacts on the progression and prognosis in NSCLC (Fig. 8). Thereupon, the newly identified interactions between FOSB and p53 may serve as potential intervention targets for NSCLC.

An abridged general view illustrating the heterogenous impacts of the transcription factor FOSB on the malignant progression and platinum-based chemotherapeutic prognosis in NSCLC with varying genetic backgrounds of *TP53* via selectively transcriptionally activating distinct target genes.

### Electronic supplementary material

Below is the link to the electronic supplementary material.

Supplementary Material 1

## Data Availability

The datasets used and/or analyzed during the current study are available from the corresponding author on reasonable request.

## Abbreviations

AKR1C3: Aldo-Keto Reductase Family 1 Member C3
ALDH3A1: Aldehyde Dehydrogenase 3 Family Member A1
AP-1: Activator protein-1
CCK-8: Cell Counting Kit-8
CDX: Cell line-derived xenograft
CENP-A: Centromere Protein A
ChIP: Chromatin immunoprecipitation
Co-IP: Co-immunoprecipitation
DBD: DNA binding domain
DEGs: Differentially expressed genes
FOSB: FBJ Murine Osteosarcoma Viral Oncogene Homolog B
GO: Gene Ontology
GOF: Gain-of-Function
IGF1R: Insulin Like Growth Factor 1 Receptor
IGFBP5: Insulin Like Growth Factor Binding Protein 5
LUAD: Lung adenocarcinoma
LUSC: Lung squamous cell carcinoma
NEDD8: Neural Precursor Cell Expressed, Developmentally Down-Regulated 8
NSCLC: Non-small cell lung cancer
OD: Optical density
OS: Overall survival
PREX1: Phosphatidylinositol-3,4,5-Trisphosphate Dependent Rac Exchange Factor 1 RAC1:Rac Family Small GTPase 1
RT-qPCR: Real-time quantitative PCR
si-RNAs: Small interfering RNAs
TCGA: The Cancer Genome Atlas
TP53: Tumor Protein P53
