# Supplementary material for: Two-polarized roles of transcription factor FOSB in lung cancer progression and prognosis: dependent on p53 status
Source: J Exp Clin Cancer Res. 2024 Aug 21;43:237. doi: 10.1186/s13046-024-03161-1 (PMC11337850; doi:10.1186/s13046-024-03161-1)
Supplement: Supplementary file 1 — Supplementary Material 1 [file 13046_2024_3161_MOESM1_ESM.docx]

Figure S1


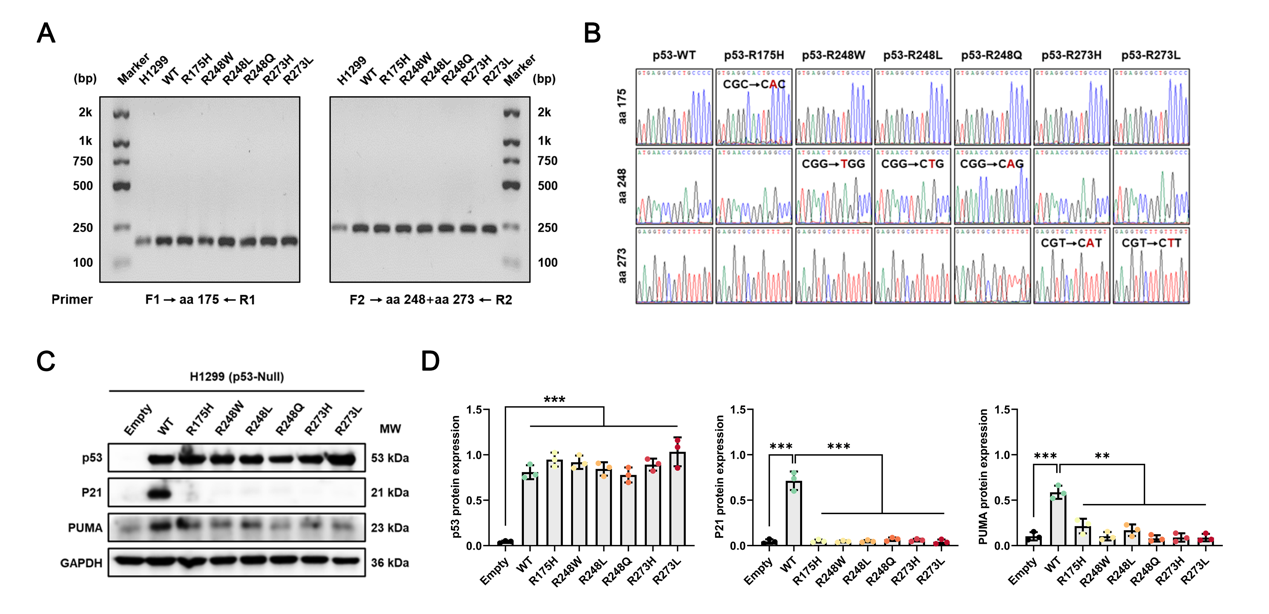


**Fig. S1 Construction of NSCLC cells ectopically expressing p53 in a range of different statuses**

(**A**) The separation by agarose gel electrophoresis of the PCR products of the exogenously introduced *TP53* gene in H1299 cells; (**B**) The validation by Sanger sequencing of the accuracy of the different *TP53* mutation sites constructed; (**C**) The protein expression levels of different statuses of p53, P21, and PUMA in H1299 cells, detected by the Western Blot; (**D**) Quantitative analysis of the immunoreactive bands displayed in (C). ** *P*<0.01, *** *P*<0.001.

Figure S2


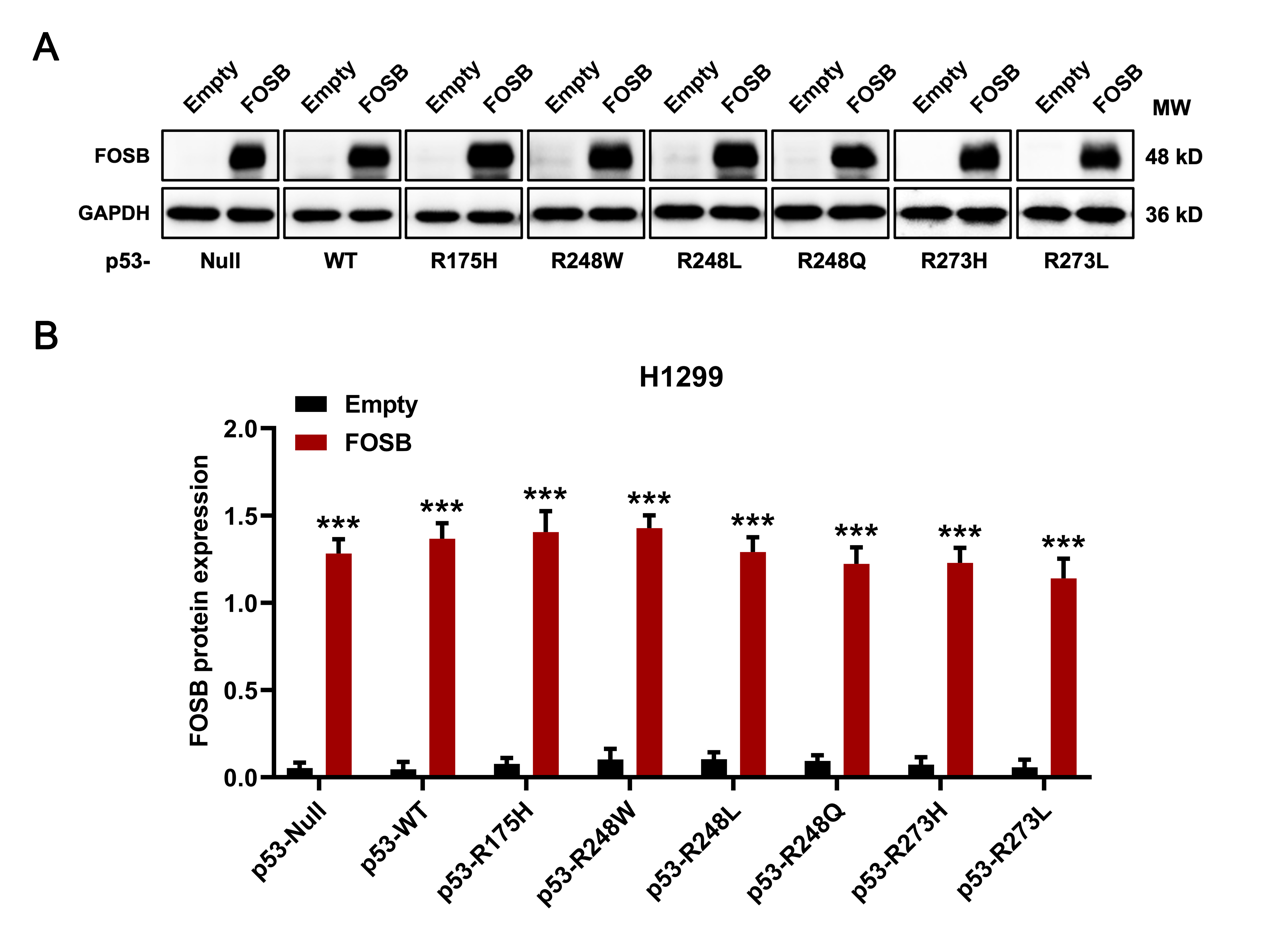


**Fig. S2 Construction of FOSB-overexpressing NSCLC cells with different genetic backgrounds of *TP53***

(**A**) The protein expression levels of FOSB in FOSB-overexpressing H1299 cells with different genetic backgrounds of *TP53*; (**B**) Quantitative analysis of the immunoreactive bands displayed in (A). *** *P*<0.001.

Table S1 Primer pairs used for the RT-qPCR

| Gene | Forward (5’-3’) | Reverse (5’-3’) |
| --- | --- | --- |
| *FOSB* | AGCTAAATGCAGGAACCGG | ACCAGCACAAACTCCAGAC |
| *TP53* | AGTCACAGCACATGACGGAG | CTCATAGGGCACCACCACAC |
| *LBH* | GCCCCGACTATCTGAGATCG | GCGGTCAAAATCTGACGGGT |
| *PREX1* | AGGCTACCTGTTGTCTCCGA | GAGCAAACGGTCTTCATGGC |
| *PTGER4* | CCGGCGGTGATGTTCATCTT | CCCACATACCAGCGTGTAGAA |
| *METTL7A* | GCGGGAGCTCTTCAGTAACC | TCTCAAAGTTGGGGTTGGGG |
| *ZBED6* | GAAGGGTTTGCGAATTAAGGGG | GGGTCATTGGAAGCTAACAAAGC |
| *IGFBP5* | CCCAATTGTGACCGCAAAGG | GGCAGCTTCATCCCGTACTT |
| *RARB* | CCCCAGAACAAGACACCATGA | TTTTGTCGGTTCCTCAAGGTC |
| *ALDH3A1* | AAGAGGAGATCTTCGGGCCT | TAATCACCTTGTCGTTGCTGGA |
| *TNFAIP2* | TGCTCCAGAACCTGCATGAGGA | AACTCAGGCAGCTCTGTGACTA |
| *AKR1C3* | GGGATCTCAACGAGACAAACG | AAAGGACTGGGTCCTCCAAGA |
| *PAPPA* | GACCCACATCCCTTTGGTATTG | CCGGTTGGGTGCTAAGGA |
| *ABCC2* | ATACCAATCCAAGCCTCTAC | GAATTGTCACCCTGTAAGAG |
| *ABCC3* | GGTCCCTAAAGGAAGAGGACAGA | ATTTTTCCCAGGTTCTGCTGAAG |
| *AKR1C1* | ATTTGCCAGCCAGGATAGTG | AGAATCAATATGGCGGAAGCC |
| *GAPDH* | CTTTGGTATCGTGGAAGGACTC | GTAGAGGCAGGGATGATGTTCT |

Table S2 Primer pairs used for the RT-PCR

| Gene | Forward (5’-3’) | Reverse (5’-3’) |
| --- | --- | --- |
| *TP53*-1 | AGTCACAGCACATGACGGAG | CTCATAGGGCACCACCACAC |
| *TP53*-2 | GGTTGGCTCTGACTGTACCA | TCCCCTTTCTTGCGGAGATTC |

Table S3 The siRNA oligo sequences used for the cell transfection

| Targets | Sense (5’-3’) | Anti-sense (5’-3’) |
| --- | --- | --- |
| si-NC | UUCUCCGAACGUGUCACGUTT | ACGUGACACGUUCGGAGAATT |
| si-PREX1 | GCGUGGGUCUGUGCAACAATT | UUGUUGCACAGACCCACGCTT |
| si-IGFBP5 | GCUGACCCAGUCCAAGUUUTT | AAACUUGGACUGGGUCAGCTT |
| si-AKR1C3 | CCGGAGUAAAUUGCUAGAUTT | AUCUAGCAAUUUACUCCGGTT |
| si-ALDH3A1 | GCAACGACAAGGUGAUUAATT | UUAAUCACCUUGUCGUUGCTT |
| si-TP53 | CUACUUCCUGAAAACAACGTT | CGUUGUUUUCAGGAAGUAGTT |

Table S4 Primer pairs used for the ChIP-qPCR

| Gene Promoter | Forward (5’-3’) | Reverse (5’-3’) |
| --- | --- | --- |
| *PREX1*-1 | CTGAATTTCCCTCCAAAAAGGC | TCCCACTACTTGTTGAATGCT |
| *PREX1*-2 | ACAGTGTGATATGGTTTGAC | TAGGTTTTCACACTGCTGCT |
| *IGFBP5*-1 | AAAAGCTACCAGTGTGCCTGA | TGAGGGCTTGGTAAGGTAGGA |
| *IGFBP5*-2 | GGTGTAACAGCCCACACCAA | AGGAACCGAATCATGCCACT |
| *AKR1C3*-1 | TTCATTTGCACCCTATTCAGGA | CTGAAGCAGTTTTGCTTTTCAGA |
| *AKR1C3*-2 | AACCATTAGGCAGTGTGCAG | CCCAGGTCAGCTTCCACTTAT |
| *ALDH3A1*-1 | TTTCGGTGACCACACAGACC | CCTTCACATCCCCCAGTGTG |
| *ALDH3A1*-2 | AGTCTGGAAAGCTGGAAGAGC | CCAGGAAGTTTGCGTGACAA |
